# Immune perturbation following SHIV infection is greater in newborn macaques than in infants

**DOI:** 10.1172/jci.insight.144448

**Published:** 2024-08-27

**Authors:** Mariya B. Shapiro, Tracy Ordonez, Shilpi Pandey, Eisa Mahyari, Kosiso Onwuzu, Jason Reed, Heather Sidener, Jeremy Smedley, Lois M. Colgin, Amanda Johnson, Anne D. Lewis, Benjamin Bimber, Jonah B. Sacha, Ann J. Hessell, Nancy L. Haigwood

**Affiliations:** 1 Department of Molecular Microbiology and Immunology, Oregon Health & Science University, Portland, Oregon, USA.; 2 Division of Pathobiology & Immunology and; 3 Genetics Division, Oregon National Primate Research Center, Oregon Health & Science University, Beaverton, Oregon, USA.; 4 Vaccine & Gene Therapy Institute, Oregon Health & Science University, Beaverton, Oregon, USA.; 5 Division of Comparative Medicine, Oregon National Primate Research Center, Oregon Health & Science University, Beaverton, Oregon, USA.

**Keywords:** AIDS/HIV, Immunology, Adaptive immunity, Innate immunity

## Abstract

Transmission of HIV-1 to newborns and infants remains high, with 130,000 new infections in 2022 in resource-limited settings. Half of HIV-infected newborns, if untreated, progress to disease and death within 2 years. While immunologic immaturity likely promotes pathogenesis and poor viral control, little is known about immune damage in newborns and infants. Here we examined pathologic, virologic, and immunologic outcomes in rhesus macaques exposed to pathogenic simian-human immunodeficiency virus (SHIV) at 1–2 weeks, defined as newborns, or at 4 months of age, considered infants. Kinetics of plasma viremia and lymph node seeding DNA were indistinguishable in newborns and infants, but levels of viral DNA in gut and lymphoid tissues 6–10 weeks after infection were significantly higher in newborns versus either infant or adult macaques. Two of 6 newborns with the highest viral seeding required euthanasia at 25 days. We observed age-dependent alterations in leukocyte subsets and gene expression. Compared with infants, newborns had stronger skewing of monocytes and CD8^+^ T cells toward differentiated subsets and little evidence of type I interferon responses by transcriptomic analyses. Thus, SHIV infection reveals distinct immunological alterations in newborn and infant macaques. These studies lay the groundwork for understanding how immune maturation affects pathogenesis in pediatric HIV-1 infection.

## Abstract 2

This study showed that defective innate defenses, elevated immune activation, and increased viral seeding are associated with age-dependent differences in SHIV pathogenesis in newborn macaques.

## Graphical abstract

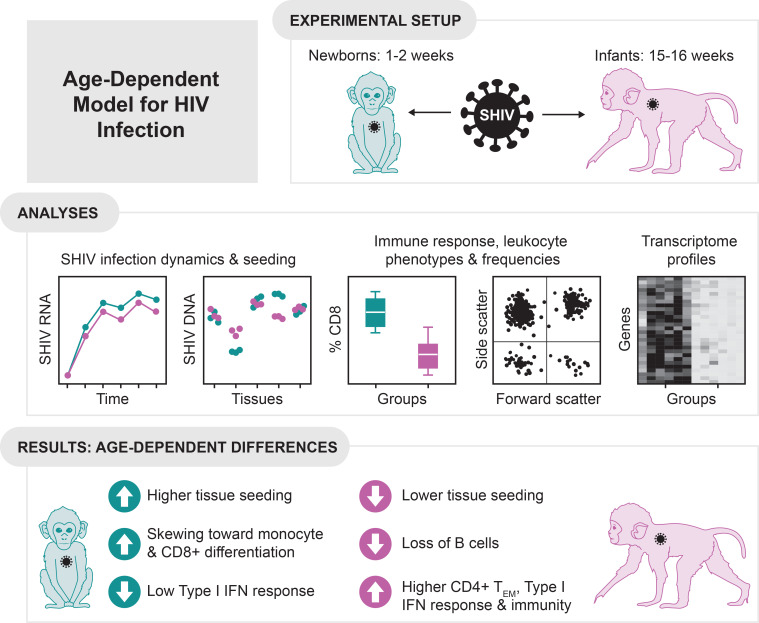

## Introduction

Vertical HIV acquisition remains prevalent in resource-limited settings, with an estimated 130,000 infants infected in 2022, because of limitations in access to antiretroviral therapy (ART) during pregnancy (1). Transmission occurs predominantly during labor and birth and also during gestation or breastfeeding (2). In newborns, untreated HIV infection is characterized by high viral loads, poor control of post-acute viremia, and rapid disease progression (3). Without treatment, over 30% of these infants die in their first year of life, and over half die by age 2 (4).

Primate lentiviral infections in humans and nonhuman primates (NHPs) show an association with a vicious cycle of chronic immune activation, impaired immunological function, and viral persistence in tissues (5). Early in infection, damage to the intestinal barrier results in microbial translocation into the blood (6). Uptake of microbial products by myeloid antigen-presenting cells stimulates pro-inflammatory cytokine secretion, leading to systemic inflammation and immune activation (7). In adults with HIV, activation and proliferation of CD8^+^ T cells occurs in a largely antigen-independent manner and is driven by pro-inflammatory cytokines, especially IL-15 (8). Studies of untreated and ART-treated HIV infection in children and adults have identified inversion of the ratio of CD4^+^ to CD8^+^ T cells as a marker of immune activation, immune senescence, and poor prognosis (9–11). Finally, alterations to the monocyte compartment during infection are associated with disease progression both in humans and in primate models (12–14). Monocytes exist on a spectrum of differentiation from classical (CD14^+^CD16^–^) through intermediate (CD14^+^CD16^+^) to nonclassical (CD14^–^CD16^+^) subsets (15, 16). Increased frequencies of intermediate monocytes correlate with systemic immune activation and negatively correlate with CD4^+^ T cell counts during HIV infection (12).

Microarray and deep-sequencing studies of gene expression have complemented these findings, identifying distinct transcriptomic signatures associated with different HIV infection outcomes. Untreated infection is characterized by an innate antiviral response mediated by type I interferon (IFN) signaling and upregulation of IFN-stimulated genes (ISGs). Even during acute HIV infection, transcriptional differences may predict the viral set point (17). Whereas rapid disease progression is marked by the upregulation of certain microRNAs (18), elite controllers and long-term nonprogressors have attenuated type I IFN responses during the chronic stage, among other alterations (19, 20). Similarly, NHP studies of acute infection with simian immunodeficiency virus (SIV) and simian-human immunodeficiency viruses (SHIV) have revealed inflammasome activation, type I IFN, and IFN-γ responses in tissues within days of mucosal exposure (21, 22). Pathogenic SIV infection drives sustained type I IFN responses during chronic infection (23), mirroring findings in human HIV-1 controllers.

Neonatal immunity is distinct from that of adults in ways that are presumed to hinder newborns’ ability to control viral infection. Newborns have greater Treg-mediated suppression and Th2 bias, deficiency in Th1 responses, underdeveloped humoral immunity, and impairments in T cell priming and other innate effector functions (24, 25). CD8^+^ T cell responses are a major mechanism of primate lentiviral control in both human and NHP studies (26). Weak CD8^+^ T cell responses are observed in HIV-infected children under 3 years of age, especially those with depleted or phenotypically altered CD4^+^ T cells in conjunction with high viral loads (27). Tregs, which are abundant in newborns, are conjectured to play multifactorial and opposing roles in HIV-1 infection by suppressing virus-specific CD8^+^ T cell responses, deleterious immune hyperactivation, or both (28). Although the relationship between immune activation and pathogenesis in infants is not well characterized, CD8^+^ T cell activation in infants at 1–2 months of age was predictive of disease progression in one study (29). Newborn and infant rhesus macaques also have greater counts and frequencies of CD4^+^ T cells (30), notably activated CD4^+^CCR5^+^ memory T cells that are prime targets for infection with SIV (31, 32) and may be a key target cell population during vertical HIV acquisition (33). Among infants who acquired HIV vertically, age at the time of infection is correlated with survival rate, with mortality highest among those infected before 4 weeks of age and decreasing gradually over the first year of life (4, 34, 35). This decrease in mortality may be attributable at least in part to immune maturation resulting in increased antiviral defense.

In adult macaques exposed to the pathogenic tier 2 viral swarm SHIV_SF162P3_, peak viremia decreases and varies between individuals (36, 37), in some cases to undetectable levels (38). In adult macaques infected with this SHIV using a high-dose mucosal (intrarectal) challenge, post-acute plasma viremia averaged 10^6^ copies/mL for the first 9 weeks of infection and 3.2 log_10_ DNA copies per microgram in PBMC and lymphoid tissues (39). In contrast, infant macaques infected with SHIV_SF162P3_ at week 4 (28 days) recapitulated the high viral loads (post-acute, persistent viremia averaging 10^7^ copies/mL) and experienced rapid disease progression seen in vertical HIV infection; seeding in PBMC and lymphoid tissues averaged 4 log_10_ copies per microgram (40–43). Many NHP studies intended to model peripartum HIV infection have assigned macaques that are several weeks (40–43) to several months old (44). In healthy rhesus macaques, immune cell populations and cytokine responses differ from adult values and evolve with age during the first weeks and months of life (45), underscoring the importance of understanding age in interpreting infant macaque studies of pediatric immunity and immunopathology.

Here, we asked whether SHIV infection dynamics, immune responses, and outcomes in infant macaques are influenced by age at the time of exposure. We compared disease progression and virologic outcomes, adaptive immune responses, frequencies and phenotypes of key leukocyte subsets, and transcriptome profiles during pathogenic SHIV infection in newborn (1–2 weeks old) and infant (15–16 weeks old) rhesus macaques that were lacking the 2 major MHC class I (MHC-I) alleles for post-acute viral control (46, 47). Viral DNA copies in tissues were compared with an out-group of adult rhesus macaques mucosally infected with the same virus for the same length of time (39). These longitudinal data highlight age-dependent quantitative and qualitative differences both in viral seeding and in the developing infant macaque immune system, providing a baseline for comparison to model the effect of novel HIV therapies on viral pathogenesis in the pediatric setting.

## Results

### Study design.

**Figure 1:**
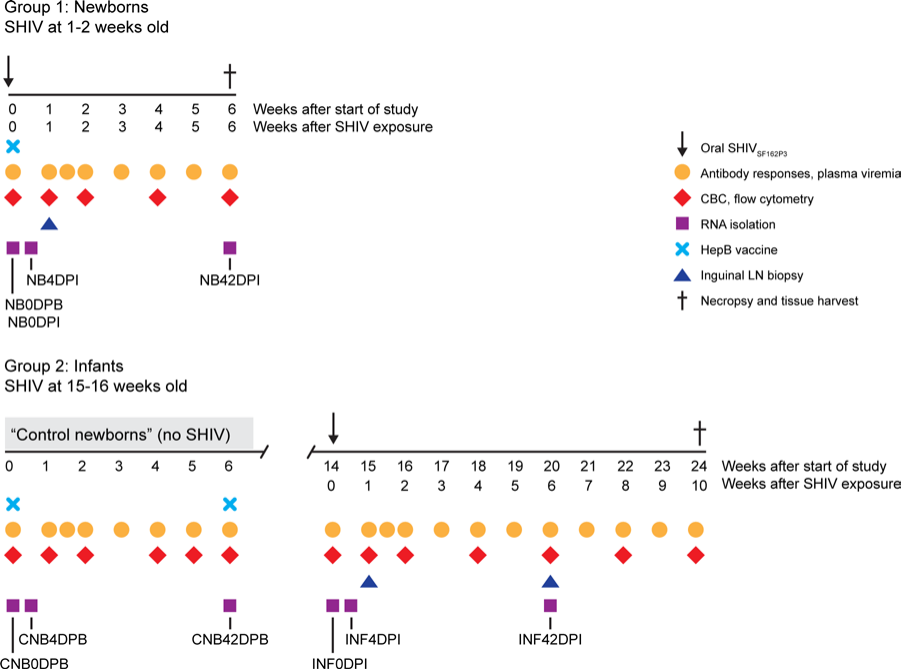
Study design. Group 1 (Newborns, *n* = 6 animals) were orally exposed to a single high dose of SHIV_SF162P3_ at 1–2 weeks of age, while those in group 2 (Infants, *n* = 6 animals) were exposed at 15–16 weeks of age. During the first 6 weeks of the study, the group 1 infants served as uninfected age-matched controls for comparison with newborns and are termed Control Newborns. All animals were given hepatitis B (HepB) vaccine (light blue X) on the first day of the study, and, for infants, a second dose was given 6 weeks later. Blood was sampled at the time points indicated in order to measure antibody responses and plasma viral loads (orange circles), complete blood counts and leukocyte phenotyping by flow cytometry (red diamonds), and RNA isolation for transcriptome analysis (purple squares). Inguinal lymph node biopsies (blue triangles) were taken at 1 week and, for infants, 6 weeks after SHIV exposure. Animals were euthanized at the time points indicated (dagger) after SHIV exposure, or sooner if clinical endpoints were met because of onset of SHIV disease, and tissues were harvested for viral load quantitation and measurement of T cell responses. Time points in the study were assigned specific labels to indicate newborns (NB), control newborns (CNB), and infants (INF); time point labels indicate days post-beginning of the study (DPB) or days post-infection (DPI).

Twelve animals were assigned to the study, regardless of sex, at approximately 1 week of age. All animals had maternal antibodies against rhesus CMV before SHIV infection, but titers waned to below detection in 6 weeks, consistent with lack of infection (data not shown). Groups of 6 rhesus macaques each were exposed to a single oral high dose (4.1 + 10^4^ tissue culture ID_50_; TCID_50_) of SHIV_SF162P3_ known to infect 100% of controls (42) during different stages of infancy (Figure 1). Animals in group 1 (newborns) were exposed to SHIV at 1–2 weeks (7–14 days) of age, while those in group 2 (infants) were exposed at 15–16 weeks of age (Table 1). Infants were allowed to age to 10 weeks after SHIV (24 weeks of age) to allow for observation of changes in viremia. Blood and lymph node biopsies were sampled at age- or infection-matched intervals during the first 6 weeks of SHIV infection. Newborns assigned to age into infants in group 2 served as age-matched uninfected controls for group 1, and blood was collected from group 2 infants at age-matched time points during the newborn period (<8 weeks old) to provide baseline measurements for comparison with the SHIV-infected newborns. We categorized these samples as control newborns. All animals were immunized with a HepB vaccine using a regimen modeled after the routine HepB immunization schedule for infants in the United States (48). Lymphoid and gastrointestinal tract tissues were harvested at necropsy for analyses of cellular composition and viral reservoir quantification.

### SHIV disease, infection dynamics, and reservoir seeding.

Two newborns (1 male, 1 female) experienced very rapid weight loss and diarrhea, accompanied by lymphodepletion, and were euthanized as required by the criteria specified in the animal care protocol on day 24 after SHIV exposure (Table 1). The other newborns and 5/6 infants survived to the experimental endpoint of 6 or 10 weeks, respectively. Clinical history was evaluated by assigning a disease score of 0–2 to indicate disease severity. In both the newborn and infant groups, 5/6 animals developed some degree of renal glomerular change or rash (disease score 1), with 2/6 newborns and 4/6 infants exhibiting lesions or opportunistic infections directly attributable to SHIV infection (disease score 2) (Table 1). Although there was no significant difference in disease score between groups (Mann Whitney *U* test, 2-tailed, *P* = 0.53; *n* = 6 animals per group), rapid progression prior to 6 weeks was observed only in the newborn group (*P* = 0.0307, Mantel-Cox test; *P* = 0.0247, Gehan, Breslow, Wilcoxon’s test, *n* = 6 per group).

**Figure 2:**
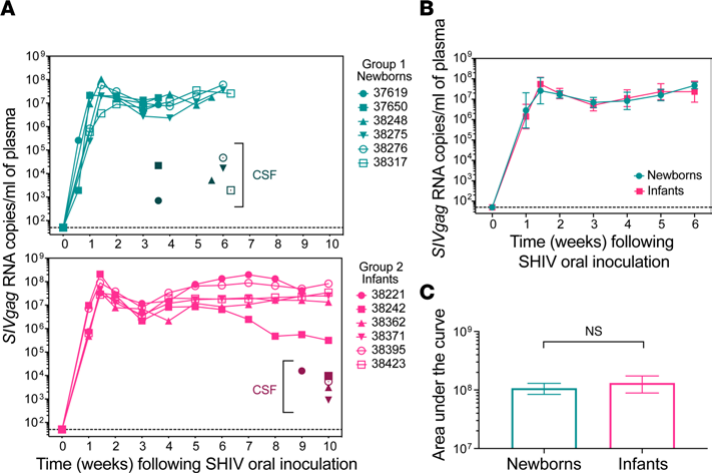
Viral loads during the acute phase following high-dose challenge with SHIV do not vary by age at time of exposure in infant macaques. (**A**) Top, plasma viral load (PVL) data for newborns (exposed to SHIV_SF162P3_ at 1–2 weeks of age). Bottom, PVL data for infants (exposed to SHIV_SF162P3_ at 15–16 weeks of age). Individual animals are represented by unique symbols. Symbols with darker colors indicate viral load in cerebrospinal fluid (CSF) at necropsy. (**B**) PVL between newborns and infants, with data censored at 6 weeks and time points excluded from analysis if PVL was measured for only 1 group. Data represent mean ± SEM. (**C**) Area under the curve was computed by taking the mean PVL measurement at each time point during the first 6 weeks after SHIV exposure, resulting in no significant difference between the groups (unpaired *t* test with Welch’s correction, 2-tailed *P* > 0.05, *n* = 6 animals per group).

We measured viral RNA copies in plasma over time (Figure 2). All animals in both age groups had high peak viremia and maintained high post-acute viral loads through week 6 after infection (p.i.), with only a transient decrease in viremia from the peak level on day 10 p.i. (Figure 2A). Animal 38242 in the infant group had reduced plasma viral load at 8–10 weeks p.i., but during the first 6 weeks, there was no significant difference in cumulative plasma viral loads between newborns and infants (Figure 2, B and C).

**Figure 3:**
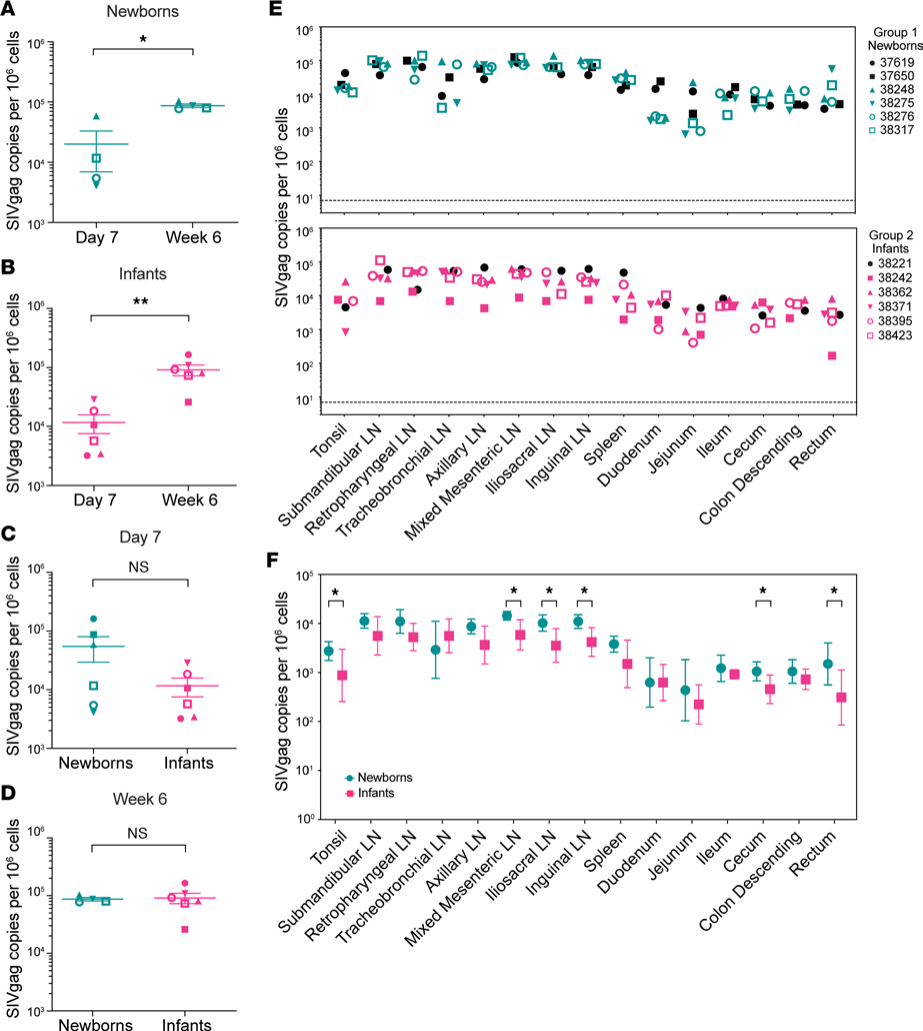
Viral DNA in tissues of newborns and infants. (**A**–**D**) Viral DNA copies in inguinal LNs from newborns and infants were compared at day 7 and week 6 using 1-way ANOVA with Tukey’s multiple comparisons. Two newborns euthanized before week 6 were excluded from **A** and **D** because there was no 6-week sample for comparison. (**A** and **B**) Newborns and infants both had significantly more viral DNA at week 6 compared with day 7 after SHIV exposure. Newborns: *P* = 0.007. Infants: *P* = 0.0005. (**C** and **D**) Viral DNA copies in inguinal LNs were not significantly different between age groups at day 7 or week 6 after SHIV exposure. Day 7: *P* = 0.756. Week 6: *P* = 0.998. (**E**) Viral DNA quantitation in lymphoid and gut tissues at time of death including all animals in the study; black symbols indicate animals euthanized at week 3 (newborns) and week 9 (infants). Newborns had a median of 0.36 logs more viral DNA copies per 10^6^ cells in each tissue than infants (Wilcoxon’s matched pairs signed-rank test on log_10_-transformed data, 2-tailed *P* = 0.0006). (**F**) Means ± SEM of individual tissues for newborns and infants (2-tailed unpaired *t* test, Benjamini, Krieger, and Yekutieli 2-stage step-up with *P* values adjusted for multiple comparison by the FDR approach). **, *P* < 0.01. *, *P* < 0.05. Symbols used for each animal are consistent throughout the manuscript.

To compare seeding rates early in infection, we quantified viral DNA in inguinal lymph nodes (LNs) at day 7 and at week 6 p.i. and found that both newborns (that survived to week 6) and infants had significant increases (Figure 3, A and B). To determine whether levels differed between these age groups, we compared these same data (viral DNA levels in inguinal lymph nodes) on day 7 or at week 6 (Figure 3, C and D), and differences were not significant at either time point. The newborns that progressed quickly to disease had the highest DNA copy numbers in lymphoid tissues on day 7 (Figure 3C). Comparison of viral DNA in 15 lymphoid and gut tissues at necropsy revealed a small but significant 0.36-log_10_ increase in viral seeding in newborns compared with infants (Figure 3E). This difference remains significant (*P* = 0.0026) if the 2 rapidly progressing newborns necropsied early (black symbols) are removed from the analysis. Pairwise comparisons of the 15 individual tissues showed that 2 gut (cecum, rectum) and 4 lymphoid samples (iliosacral, inguinal, and mixed mesenteric LNs and tonsil) were significantly different between newborns and infants (Figure 3F). Necropsy tissue samples were collected at week 9 or 10 for infants and at week 3 or 6 for newborns (see Table 1), which are not precisely matched time points. We previously showed that, in this infant model, viral DNA levels in PBMC and lymphoid tissues peak at 6 weeks and are stable to at least 16 weeks (42). We compared DNA copies/million cells for 11 tissues (5 lymphoid and 6 gut) from this study with those from 6 adult macaques infected with SHIV_SF162P3_ and necropsied at 8 weeks after infection (39) (Supplemental Table 1; supplemental material available online with this article; https://doi.org/10.1172/jci.insight.144448DS1). Mean newborn DNA levels were significantly higher than adult levels comparing all 11 tissues (*P* = 0.0134) and a subset of 5 lymphoid tissues only (*P* = 0.0029), based on Bonferroni-adjusted *P* values (Supplemental Table 2). Other comparisons were not significant.

### Adaptive immune responses.

**Figure 4:**
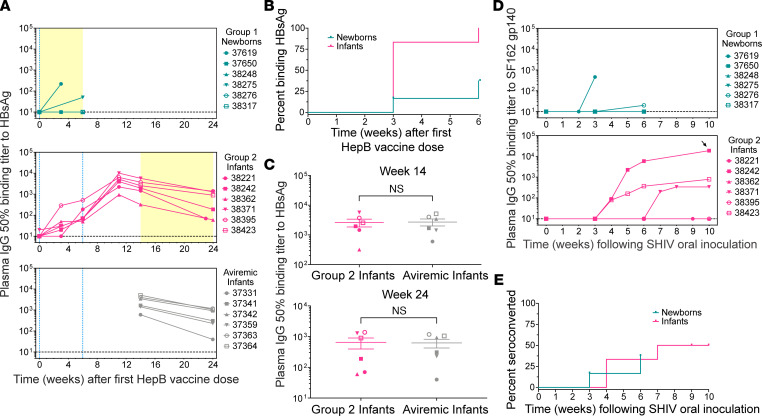
Acute SHIV infection impairs humoral responses to HepB vaccine in newborns. (**A**) Plasma IgG binding titers to hepatitis B (HepB) surface antigen recombinant protein (HBsAg) in newborns (top), infants (middle), and a comparison group of approximately age-matched infants without detectable SHIV viremia (bottom). These animals, termed aviremic infants, were exposed to SHIV_SF162P3_ on the same day as their first HepB vaccine dose, followed 30 hours later by a neutralizing antibody treatment that halted infection, resulting in undetectable viremia. Blue dotted lines at *x* = 0 and *x* = 6 denote the first and second HepB vaccine doses; newborns only received the first dose. Yellow shaded areas indicate time periods after SHIV exposure. (**B**) Kaplan-Meier analysis of the percentage of animals in each group with HBsAg antibodies. Log-rank test, *P* = 0.0115; *n* = 6 animals per group. (**C**) Comparison of HBsAg binding titers in group 2 infants and aviremic infants at either 14 weeks (top) or 24 weeks (bottom) after the first dose of HepB vaccine (unpaired *t* tests, 2-tailed *P* = 0.8225 and *P* = 0.8473 for weeks 14 and 24, respectively; *n* = 6 animals per group). Animal symbols are consistent throughout the manuscript. Data represent mean ± SEM. (**D**) Plasma IgG binding titers to HIV-SF162 Env gp140 glycoprotein in newborns (top) and infants (bottom). Black arrow indicates the only sample with neutralizing activity against SHIV_SF162P3_ (ID_50_ = 1,200). (**E**) Kaplan-Meier analysis of the percentage of animals that developed binding antibodies to SF162 gp140 by the indicated time points. Log-rank test, *P* = 0.8354, *n* = 6 animals per group.

HepB vaccination served as a measure of adaptive responsiveness (Figure 4A). All 6 infants made antibodies against surface antigen HBsAg after their first vaccine dose, with 5/6 (83%) responding by week 3, while 4/6 newborns did not respond (Figure 4B). We measured the effect of SHIV on preexisting HepB immunity by comparing the SHIV-infected infants with an aviremic control group (Figure 4, A and C) that were matched to the infants by age and HepB vaccination history (42). HepB antibody titers in these 2 groups were similar, suggesting that persistent SHIV viremia did not diminish preexisting antibody immunity to HepB in infants (Figure 4C).

**Figure 5:**
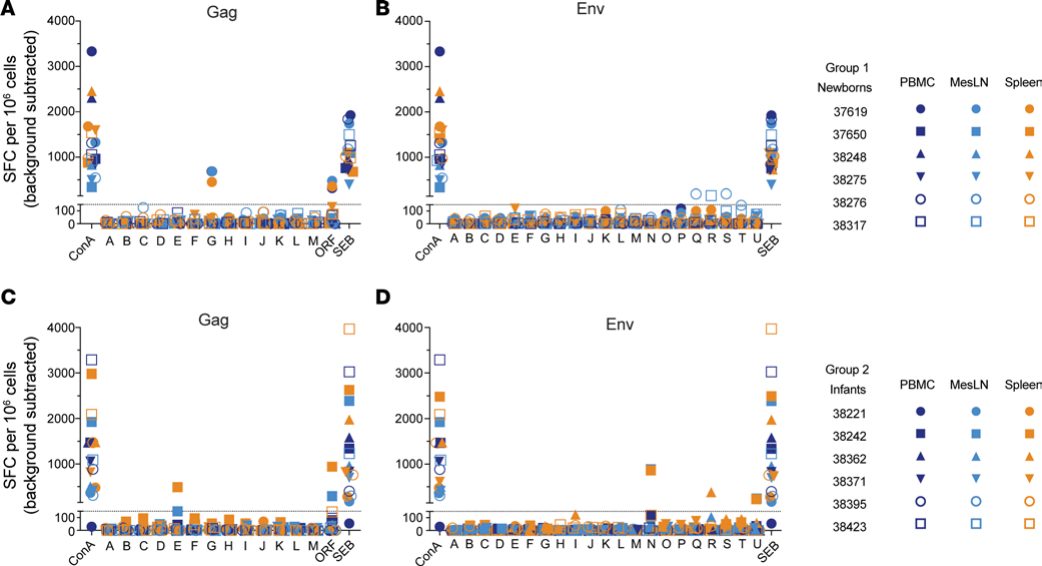
T cell responses to Gag and Env peptide pools. T cell responses to SIVmac239 Gag and Clade B consensus Env 15-mer peptide pools were measured in PBMCs by IFN-γ ELISPOT necropsy in newborns (**A** and **B**) and in infants (**C** and **D**). Symbol shapes in the key correspond to individual animals and colors indicate tissue type. Concanavalin A (ConA) and/or staphylococcal enterotoxin B (SEB) were used as positive stimulation controls. Background signal was determined using no-antigen controls. MesLN, mixed mesenteric lymph nodes; SFC, spot-forming centers.

Seroconversion to the infecting SHIV was observed in 2/6 (33%) newborns and 3/6 (50%) infants (Figure 4D). Among the newborns, 37619 seroconverted by week 3 but did not survive beyond day 25; all other animals remained negative except for 38276, which had titers just above the limit of detection at week 6 (Figure 4D). Seroconverting infants had stronger responses, with 38242 reaching titers of 10^4^ by week 6 and developing moderate neutralizing activity by week 10 (Figure 4D). Time to seroconversion was similar among newborns and infants (Figure 4E). T cell responses were measured in PBMCs, mesenteric LN, and spleen, using pools of SIV_mac239_ Gag and HIV-1 Clade B consensus Env peptides by IFN-γ ELISPOT, and responses were weak or absent in newborns (Figure 5, A and B) and infants (Figure 5, C and D). Newborn 37619 and infant 38242 had weak and narrowly focused responses on just 1 or 2 peptide pools. A weak positive signal was also observed for infant 38362 in the spleen, though not in LN and PBMCs, against 1 Env peptide pool (Figure 5D). However, the possibility of T cells recognizing other SHIV epitopes or producing other cytokines cannot be ruled out; indeed, HIV-specific CD8^+^ T cells have been found to produce low IFN-γ levels in adult women with acute infection (49), suggesting that IFN-γ secretion may not be a definitive indicator for HIV-specific CD8^+^ T cell responses.

### Leukocyte dynamics in peripheral blood.

To probe the effects of SHIV infection on major immune cell populations, we performed longitudinal flow cytometric analyses of peripheral blood leukocytes. One staining panel was designed to enumerate B cells, natural killer (NK) cells, natural killer T (NKT) cells, and monocyte subsets (Supplemental Figure 1). A second panel tracked naive and memory T cell subsets and their activation marker phenotypes (Supplemental Figure 2). A complete blood count for each sample facilitated the quantitation of absolute cell counts from flow percentages. To analyze differences due to SHIV infection status in the newborn period, data for newborns and infants were aligned according to time after the start of the study, such that animals were matched in age, but only the newborns had been inoculated with SHIV. Conversely, to determine the impact of age at SHIV exposure on immune subsets, the 2 groups were instead aligned according to time after SHIV exposure. For each immune parameter, the groups were compared at each time point using Holm-Šídák multiple comparisons test to examine differences due to age or SHIV.

There were no significant differences in absolute counts of white blood cells, neutrophils, lymphocytes, B cells, T cells, or monocytes between groups during either the first 6 weeks of the study or the first 6 weeks of SHIV infection (Supplemental Figure 3, A–C, E, F, and H). In these groups our observations included the following. (i) Platelet and NKT cell counts in newborns were marginally higher than in infants on day 4 after SHIV exposure (Supplemental Figure 3, D and G). (ii) B cell numbers gradually increased in both groups during the first 6 weeks (Supplemental Figure 3E, top), consistent with previous findings in both human and rhesus macaque newborns (30, 50). (iii) Although B cell phenotypes were not examined in detail, total B cell counts dropped during acute infection in infants but not newborns (Supplemental Figure 3E, bottom) and may reflect the preferential loss of memory B cells (51, 52), which are more abundant in human infants than in newborns (50).

NK cell counts among newborns were highly variable, obscuring differences between infected and uninfected newborns (Supplemental Figure 3I, top), but at day 10 p.i. NK cell counts dipped transiently to fewer than 200 NK cells/μL blood in both infants and newborns (Supplemental Figure 3I, bottom). Here, we provisionally refer to CD3^–^CD8^+^CD20^dim^ cells as CD20^dim^ NK cells and noted lower levels of these cells in newborns than infants just before SHIV infection and during acute infection, and levels were similar in SHIV-infected and control newborns (Supplemental Figure 3J). In general, fewer CD20^dim^ NK cells expressed CD16^+^ than CD20^–^ NK cells, but CD16 expression was similar among all animals (Supplemental Figure 3K) except at week 4, when higher levels of CD16 were observed in SHIV-infected newborns compared with control newborns (Supplemental Figure 3L, top). The implications of these differences are unclear and merit further study.

**Figure 6:**
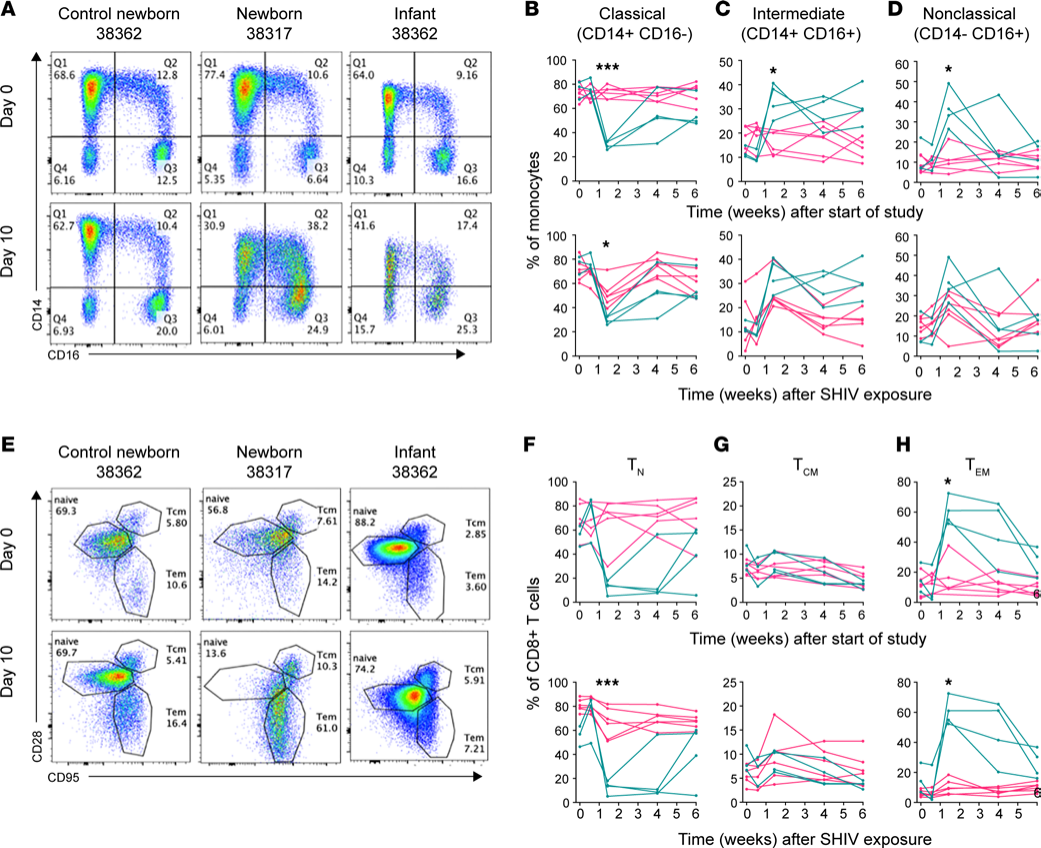
Newborn CD8^+^ T cells and monocytes are strongly skewed toward differentiated phenotypes during acute SHIV infection. Monocytes and CD8^+^ T cells were monitored longitudinally in peripheral blood by flow cytometry. (**A**) Within the CD20^–^CD3^–^CD8^–^ population, classical (Q1), intermediate (Q2), and nonclassical (Q3) monocyte subsets were resolved by expression of CD14 and CD16. Percentages of monocytes with a (**B**) classical phenotype (CD14^+^CD16^–^), (**C**) intermediate phenotype (CD14^+^CD16^+^), and (**D**) nonclassical phenotype (CD14^–^CD16^+^). (**E**) Within the CD3^+^CD4^–^CD8^+^ population, naive (T_N_), central memory (T_CM_), and effector memory (T_EM_) CD8^+^ T cell subsets were resolved by expression of CD28 and CD95. Percentages of CD8^+^ T cells with a (**F**) naive (CD28^+^CD95^–^), (**G**) central memory (CD28^+^CD95^+^), and (**H**) effector memory (CD28^–^CD95^+^) phenotype. Newborns are shown in teal and infants/control newborns in pink. Representative plots are shown from newborns and infants just before and on day 10 of SHIV infection and from control newborns on study days 0 and 10. In each set, the top graph shows weeks after the beginning of the study, where differences between the groups are due to SHIV infection status (teal: SHIV^+^ newborns, pink: SHIV^–^ control newborns). The bottom graph shows measurements after SHIV infection, where differences between the groups are due to age at the time of SHIV exposure (teal: 1–2 weeks, pink: 15–16 weeks). Pairwise statistical comparisons at each time point were performed using Holm-Šídák multiple comparisons test. *, *P* < 0.05. ***, *P* < 0.001.

Consistent with a previous study (13), absolute monocyte counts were largely stable regardless of SHIV infection or age (Supplemental Figure 3H). However, the relative frequencies of classical, intermediate, and nonclassical monocytes were altered in both newborns and infants during acute SHIV infection (Figure 6A). The proportion of classical monocytes was significantly reduced on day 10 of SHIV infection in newborns yet was unchanged in control newborns (Figure 6B). The magnitude of this reduction was greater in newborns than in infants at the same time point after SHIV exposure (Figure 6B), suggesting an age-dependent difference in the impact of SHIV on monocyte differentiation. The decrease in classical monocytes accompanied increases in both intermediate and nonclassical monocytes (Figure 6, C and D) during SHIV infection in both newborns and infants. Neither intermediate nor nonclassical monocytes differed significantly in frequency based on age at the time of SHIV exposure (Figure 6, C and D).

We found no alterations in absolute CD4^+^ and CD8^+^ T cell counts in either newborn or infant macaques (Supplemental Figure 4, A and B) and with no CD4/CD8 ratio inversion at 2–3 weeks as reported in adult rhesus macaques after vaginal infection with SHIV_SF162P3_ (38) (Supplemental Figure 4C). However, the CD4/CD8 ratio decreased in newborns at weeks 4 and 6 after SHIV exposure compared with control newborns, consistent with a study showing that CD4/CD8 ratios in human infants vertically infected with HIV-1 gradually decline from normal levels of greater than 2 but remain greater than 1 during the first year of life (53).

To track CD4^+^ and CD8^+^ T cell subset dynamics, we defined naive T (T_N_), central memory T (T_CM_), and effector memory T (T_EM_) cells based on differential surface expression of CD28 and CD95 (54). We found that the CD4^+^ compartment was predominantly naive in all animals regardless of infection, although infants had somewhat lower CD4^+^ T_N_ frequencies than newborns 6 weeks p.i. (Supplemental Figure 4D). T_CM_ comprised approximately 10%–20% of CD4^+^ T cells in control newborns, and their frequency decreased gradually during SHIV infection in both newborns and infants (Supplemental Figure 4E). In contrast, CD4^+^ T_EM_ cells — the smallest subset in control newborns — increased significantly during SHIV infection in infants, but not newborns, with significant differences between groups evident as early as day 4 p.i. (Supplemental Figure 4F).

Unlike the relatively modest changes in the CD4^+^ compartment, CD8^+^ T cell naive and memory subsets were strongly altered during SHIV infection (Figure 6E). As previously reported (54), the majority of CD8^+^ T cells were naive in the absence of infection (Figure 6F). However, in newborns but not infants, SHIV infection heavily skewed the CD8^+^ T cell compartment away from naive and toward effector memory, inverting the T_N_/T_EM_ ratio by day 10 p.i. (Figure 6, F and H). The magnitude of these rapid changes was maintained in most newborns through week 4 and was still altered at week 6 p.i. No changes in the frequencies of CD8^+^ T_CM_ were noted (Figure 6G).

The striking observation that acute SHIV infection causes profound expansion of CD8^+^ T_EM_ in newborns, but not infants, led us to ask whether immune activation phenotypes differ between age groups. We monitored expression of the activation markers CD69, CD25, and HLA-DR on the surface of naive, central memory, and effector memory CD4^+^ and CD8^+^ T cells. Surprisingly, SHIV infection did not result in overall increased expression of any of these activation markers on most T cell subsets (Supplemental Figure 4, G–X).

### Cumulative differences in blood leukocyte phenotypes.

To further analyze immunological differences between newborns and infants, we calculated the mean area under the curve (AUC) for each parameter measured longitudinally by flow cytometry during the first 6 weeks of the study as well as the first 6 weeks of SHIV infection. For each parameter, AUCs in newborns and infants were directly compared using *t* tests, with separate analyses done to test for differences during the first 6 weeks of the study (SHIV-infected newborns versus control newborns) and the first 6 weeks of infection (newborns versus infants), and significance was determined using a false discovery rate (FDR) approach. If AUCs for a parameter differed significantly between SHIV newborns and control newborns during the first 6 weeks of the study, the difference was attributed to SHIV infection status because the groups were matched in age. Conversely, if AUCs differed significantly between newborns and infants during the first 6 weeks of SHIV infection, the difference was attributed to age at the time of SHIV exposure.

**Figure 7:**
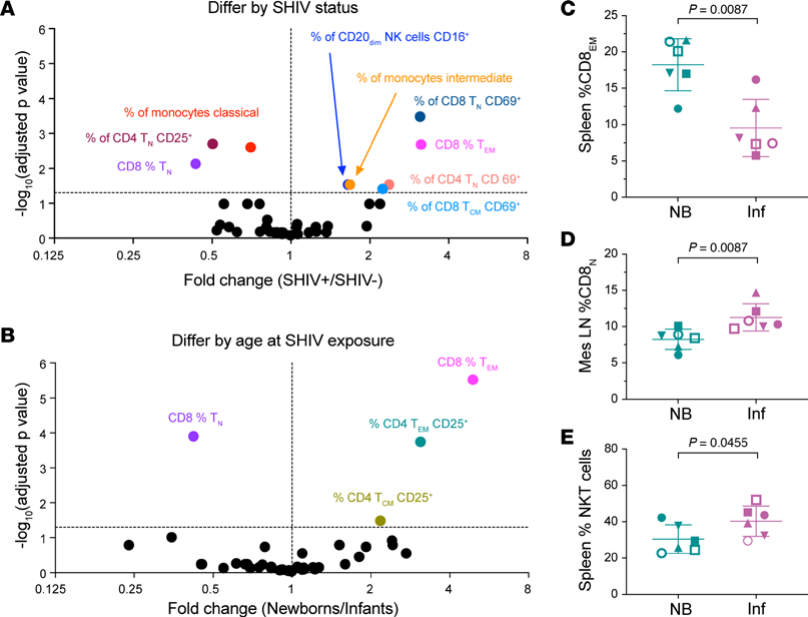
Between-group differences in leukocyte counts and phenotypes during SHIV infection in early life. (**A**) For each of 42 parameters measured by flow cytometry, AUC during the first 6 weeks of the study was computed for each group. Groups were compared by 1 *t* test per AUC comparison (total 2-tailed *t* tests: 42); *P* values were adjusted for multiple comparisons by the FDR approach. (**B**) AUC during the first 6 weeks of SHIV infection was computed for each group, using 1 *t* test per AUC comparison (total 2-tailed *t* tests: 42); *P* values were adjusted for multiple comparisons by the FDR approach. Differences were determined using the 2-stage linear step-up procedure of Benjamini, Krieger, and Yekutieli, *q* = 0.05. Each parameter was analyzed individually, without assuming a consistent SD. Differences are denoted by colored symbols and corresponding labels. In each plot, the horizontal line indicates the –log_10_-transformed threshold of significance at the *q* = 0.05 level. Statistically significantly different cell types in spleen and mesenteric LNs collected at necropsy include (**C**) percentage CD8^+^ effector memory in the spleen, (**D**) percentage CD8^+^ naive in the mesenteric LNs, and (**E**) percentage NKT cells in the spleen. *P* values are shown on each graph. Symbols identify animals as defined in Figure 2.

We identified 9 parameters for which AUCs differed significantly between groups as a function of SHIV infection status during the newborn period (Figure 7A). In line with our findings that SHIV infection skewed CD8^+^ T cell and monocyte subset frequencies, especially around day 10 p.i. (Figure 6), the cumulative percentages of classical and intermediate monocytes were 25% lower and 70% higher, respectively, in SHIV-infected newborns compared with control newborns. CD8^+^ T cells were even more strongly skewed, with >2-fold lower cumulative frequencies of CD8^+^ T_N_ and >3-fold higher frequencies of CD8^+^ T_EM_ over the first 6 weeks of SHIV infection. Interestingly, despite the paucity of significant intergroup differences in activation marker expression at any particular time point (Supplemental Figure 4, G–X), AUC analysis revealed that SHIV-infected newborns had significantly greater cumulative frequencies of CD69^+^ cells within several T cell subsets than their uninfected counterparts. In line with time point–specific comparisons, CD25^+^CD4^+^ T_N_ cell frequencies were significantly decreased during SHIV infection. Because this cell subset has been reported to express high levels of lymphoid tissue homing markers CD62L and CCR7 (55), their disappearance from peripheral blood may have been due to migration into lymphoid tissues during SHIV infection. Finally, SHIV infection was associated with a significant enrichment of CD16^+^CD20_dim_ NK cells; the biological importance of this observation is unknown.

In a parallel FDR analysis of intergroup differences during the 6 weeks after SHIV infection, we identified 4 parameters for which AUCs differed significantly between groups as a function of age at the time of SHIV exposure (Figure 7B). Importantly, 2 of these parameters — the frequencies of CD8^+^ T_N_ and CD8^+^ T_EM_ — were found to differ as a function of both SHIV infection status and age at SHIV exposure, indicating an age-dependent difference in the effect of SHIV. In addition, cumulative frequencies of CD25^+^CD4^+^ T_CM_ and T_EM_ were 2- and 3-fold greater in newborns than in infants, possibly reflecting the relatively greater abundance of Tregs in the neonatal immune system. At necropsy, we analyzed the same surface markers in spleens and mesenteric LNs to compare differences by age at time of SHIV exposure in the newborns and infants. The percentage CD8^+^ T_EM_ cells was significantly greater in the spleens of newborns (Figure 7C), and percentage of CD8^+^ T_N_ cells was greater in the mesenteric LNs of infants than in newborns (Figure 7D), consistent with the ratios seen in the blood (Figure 7B). In the spleen, the percentage of NKT cells was significantly lower in newborns than in infants (Figure 7E). Other comparisons were not significant.

### Transcriptome analysis.

**Figure 8:**
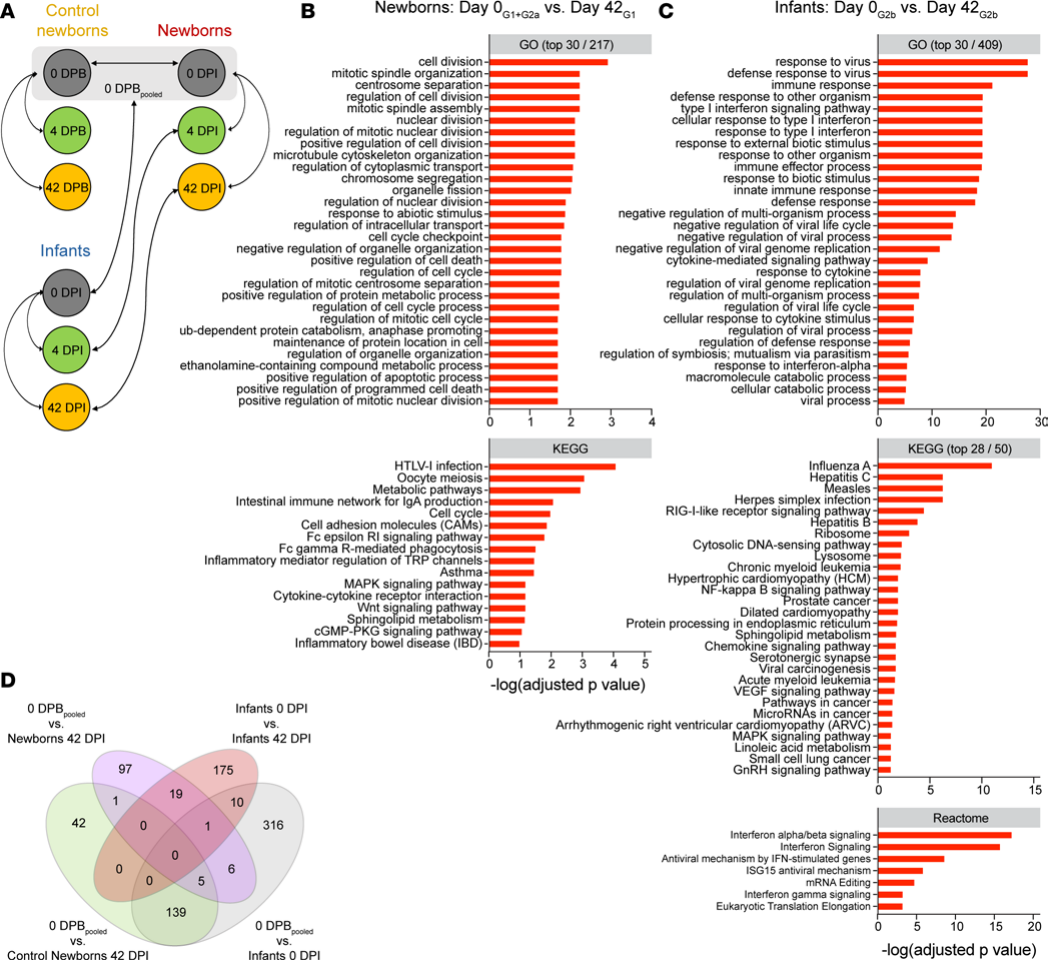
RNA-Seq reveals distinct transcriptomic signatures in newborns and infants on day 42 of SHIV infection. (**A**) Contrasts of interest are shown with arrows. 0 DPB_pooled_ is the combined sample group of all uninfected newborns at the beginning of the study (*N* = 12 animals). GO, KEGG, and Reactome analyses of differentially expressed genes in newborns (**B**) and infants (**C**) on 42 DPI versus 0 DPI (adjusted *P* < 0.1). Where many terms were significant (adjusted *P* < 0.1), top significant terms are shown. No pathways were significantly enriched in the Reactome database. (**D**) Venn diagram of differentially expressed genes in newborns 42 DPI versus 0 DPB_pooled_ (purple), infants 42 DPI versus infants 0 DPI (pink), and background transcriptional signatures in control newborns (green) and infants (gray) at different time points in the absence of SHIV infection.

To compare innate responses to SHIV infection by age, we performed transcriptome analysis in the peripheral blood using bulk RNA sequencing (RNA-Seq). For newborns and infants, we analyzed samples on day 0 just prior to SHIV exposure, as well as day 4 and (when available) day 42 of SHIV infection (0, 4, and 42 DPI). For an SHIV-unexposed, age-matched baseline for comparison with the newborns, we also included control newborn samples on days 0, 4, and 42 from the beginning of the study (0, 4, and 42 DPB) (Figure 8A). The design of our study allowed differential expression (DE) analyses to be performed across multiple contrasts, designed to measure different facets of infection (Figure 8A). Contrasts at time points before SHIV exposure measured age-dependent differences or cohort/batch effects. Within-group contrasts after SHIV exposure measured the impact of SHIV infection in newborns and in infants. Across-group contrasts at matched time points after SHIV exposure evaluated age-dependent responses to SHIV infection.

We began our analysis by examining differentially expressed genes in contrasts prior to SHIV exposure to measure baseline/background differences in gene expression. Only 3 genes were significantly differentially expressed (adjusted *P* < 0.1) between newborns on 0 DPI and control newborns on 0 DPB (Supplemental Figure 5A). Therefore, these groups were combined into a single 0 DPB_pooled_ group for subsequent contrasts. No genes were significantly differentially expressed between 0 DPB_pooled_ and control newborns 4 DPB. However, a signature of 187 differentially expressed genes was noted for control newborns at 42 DPB versus 0 DPB_pooled_ (Supplemental Figure 5E). An even stronger signature with 477 differentially expressed genes was found for infants at 0 DPI contrasted with 0 DPB_pooled_ (Supplemental Figure 5F). Given that control newborns and infants are the same animals sampled at different ages, this result is consistent with age-dependent transcriptomic changes as these animals grew older in the absence of SHIV infection. In both contrasts, Gene Ontology (GO) and Kyoto Encyclopedia of Genes and Genomes (KEGG) revealed DE of genes associated with various processes related to immune system development and signaling (Supplemental Figure 6, A and B), likely reflecting normal immunological maturation during early life.

When we evaluated within-group differences in gene expression for newborns and infants during SHIV infection, only 5 genes were found to have significant DE between 0 DPI and 4 DPI in newborns, suggesting that gene expression in peripheral blood is not yet substantially altered at this early time point in acute infection (Supplemental Figure 5B). However, by 42 DPI, we detected 129 genes with DE, of which 57 (44%) were upregulated and 72 (56%) were downregulated relative to 0 DPB_pooled_ (Supplemental Figure 5C). Similarly, for intragroup contrasts among infants, we compared gene signatures in infants 0 DPI with those 4 DPI and 42 DPI. No significant genes were identified as having DE 0 DPI versus 4 DPI. However, 42 DPI of SHIV infection in infants, 205 genes were differentially expressed, of which 158 (77%) were upregulated and 47 (23%) were downregulated relative to 0 DPI (Supplemental Figure 5D).

GO and KEGG analysis showed that the DE signature at 42 DPI in newborns consisted largely of genes involved in cell division, cell cycle regulation, and metabolism (Figure 8B). Genes in the most significantly enriched term cell division were *AURKA*, *ECT2*, *KIF23*, *KIF11*, *BUB1B*, *CCNB2*, *BIRC5*, *PRKCE*, *CDC20*, *CDCA2*, *PTTG1*, *NEK2*, *CEP55*, *ZWINT*, and *TOP2A*; most were significantly downregulated 42 DPI compared with 0 DPB_pooled_. We observed upregulation of several genes, including *TLR7*, *ISG20*, *CXCL10*, and *LIF*, which were part of the significantly enriched term immune response (adjusted *P* value = 0.048), though they did not seem to cluster with any specific pathway. CXCL10 in particular is a biomarker for high viremia and rapid HIV-1 disease progression (20, 56, 57), as well as immune dysfunction in infectious diseases more broadly (58). In infants, GO and KEGG analyses comparing day 0 versus day 42 of infection revealed differential regulation of gene networks involved in antiviral immune responses mediated by type I IFN signaling (Figure 8C). Genes contributing to enriched terms containing the phrase “type I interferon” included *ADAR*, *BST2*, *IFI6*, *IFI27*, *IFIT1*, *IFIT3*, *ISG15*, *ISG20*, *MX1*, *MX2*, *OAS1*, *OAS2*, *OAS3*, *RSAD2*, *SAMHD1*, *SP100*, *STAT2*, *USP18*, and *XAF1*. Notably, many of these are lentiviral restriction factors, and most were significantly upregulated 42 DPI. The finding that CXCL10 was significantly differentially expressed in newborns but not in infants suggests a possible role for CXCL10 in age-dependent mechanisms of SHIV pathogenesis. Newborn and infant transcriptional signatures were very distinct, with 97/129 (75%) of genes differentially expressed in newborns and 175/205 (85%) of genes differentially expressed in infants specific to each group’s SHIV response 42 DPI (Figure 8D). Of the 291 total genes having DE in either group specifically on day 42 of SHIV infection relative to that group’s 0 DPI baseline, only 19 genes (6.5%) were common to both groups (Figure 8D).

Finally, we examined between-group differences in gene expression at matched time points during SHIV infection. As previously noted, 477 genes were differentially expressed on 0 DPI in infants versus on 0 DPB_pooled_ in newborns, indicating a substantial age-dependent difference in transcriptomic profiles at baseline (Supplemental Figure 5F). By 4 DPI, only 237 genes were found to have DE in newborns versus infants, a 2-fold decrease (Supplemental Figure 5G). On 42 DPI, only 38 genes had DE, a 12.5-fold decrease from day 0 (Supplemental Figure 5H). GO and KEGG analysis of DE at 42 DPI revealed that there was no clear enrichment for particular biological processes or signaling pathways, aside from a single significant term, “metabolic pathways, found in KEGG” (Supplemental Figure 6D). GO and KEGG analyses showed different gene expression hierarchies between 0 DPI (Supplemental Figure 6B) and 4 DPI (Supplemental Figure 6C), which showed DE of genes involved in metabolism, porphyrin synthesis, and erythrocyte development. Taken together, and in light of the results from the within-group comparisons (Figure 8), these data paradoxically suggest that, although SHIV responses differ by age, SHIV infection ultimately reduces age-dependent differences in gene expression. This phenomenon is detectable as early as 4 DPI, a time point when SHIV infection has little effect on gene expression in newborns (Supplemental Figure 5B) and no significant effect in infants.

## Discussion

Without treatment, vertical transmission of HIV is likely to cause severe disease in infants with high viremia, but determinants contributing to higher viremia are not known (3). Here, we show in a macaque oral infection model that despite similar kinetics of viral seeding in blood and lymphoid tissues at day 7, 2 newborns with the highest plasma viremia succumbed to very early disease prior to the time of planned euthanasia. Although significantly higher levels of viral DNA were recorded in 15 tissues of newborns compared with infants at necropsy (Figure 3E), seeding was variable, with significant differences in 2 gut and 4 lymphoid tissues (Figure 3F). Viral DNA in all 11 tissues and in a subset of 5 lymphoid tissues was also significantly greater in newborns than in adults (39), supporting the hypothesis that a younger age at the time of infection leads to increases in the size of the reservoir in certain tissues.

All newborn macaques in this study exhibited features consistent with increased immune damage and defective antiviral innate immunity during acute infection compared with infants infected at 4 months of age. In agreement with previous studies showing impaired vaccine responses in HIV-infected children, weaker HepB antibody responses to HepB were seen in newborns infected with SHIV compared with age-matched uninfected newborns. It is probable that SHIV weakens humoral responses to other vaccine antigens, given that reductions in vaccine-induced antibody titers after 6 childhood vaccines have been reported in HIV-infected infants (59). Our data suggest that this SHIV model recapitulates this important aspect of HIV-1 pathogenesis in the pediatric setting. In contrast, we found that adaptive responses to SHIV arose with similar kinetics in infants and newborns, although the study timeline was too short to complete the normal course of HepB vaccination, and it was difficult to compare the potency of antibody responses, which typically take months to reach a plateau.

Monocyte and T cell compartments were markedly altered in the blood during SHIV infection, especially in newborns. First, SHIV infection skewed monocytes toward intermediate and nonclassical phenotypes, and this skewing was more pronounced in newborns than in infants. We also detected profound alterations in T cell subsets, with an inverted ratio of naive to effector memory CD8^+^ T cells in both age groups. Analyses of leukocyte phenotype frequencies in splenic and mesenteric LN tissues at the time of necropsy revealed similar findings for CD8^+^ T_EM_ and CD8^+^ T_N_ cells in newborns and infants. Though we did not determine what proportion of these CD8^+^ T_EM_ cells were specific to SHIV epitopes, T cell responses to Gag and Env in most animals in both groups were essentially negative, suggesting that this increase reflected antigen-independent activation and expansion of CD8^+^ T cells as seen in adults with HIV (8). The age-dependent increases in differentiated monocytes and CD8^+^ T cells in newborns may be indicative of greater levels of systemic immune activation. Unexpectedly, we did not detect clear age- or infection-dependent differences in activation marker expression on T cell subsets, in contrast with studies documenting HLA-DR upregulation on CD8^+^ T cells during acute HIV-1 infection in adults and infants (29, 49). It is possible that other measures of systemic immune activation would have been informative; for instance, soluble CD14 is secreted by monocytes and macrophages stimulated by LPS and is a biomarker of microbial translocation resulting from gut epithelial barrier damage (7).

Stark differences in transcriptomic signatures were associated with SHIV infection in newborns and infants, raising the possibility that antiviral immunity is impaired in newborns due in part to underdeveloped type I IFN responses, which were intact and readily detectable in infants infected at 15–16 weeks of age. In contrast, newborns had a transcriptional signature dominated by the downregulation of genes involved in mitotic, cell cycle, and activation processes; the near absence of a viral infection response; and upregulation of *CXCL10*, a biomarker of greater disease severity. Given the central importance of type I IFN signaling in mounting an effective innate antiviral defense during acute infection (22, 60), its presence in 4-month-old macaques yet profound lack in newborns may explain, in part, the age-dependent difference in rapid disease in this model as well as pathogenesis seen in children infected with HIV-1 in utero or at birth compared with those infected at older ages. The timing of the development of type I IFN–mediated immune function during infancy should be defined more precisely in future studies, given evidence that type I IFN may contribute to rapid progression in SIV-infected infants (61) and that resolution of innate activation is associated with lack of pathogenesis in adapted hosts (23, 62).

We also examined the relationship between immune maturation over the course of early infancy and damage associated with pathogenic lentiviral infection. The strengths of our study lie in the comprehensive immunologic and transcriptomic profiling, as well as in the study design and protocol with attention to age-matched controls. Second, to our knowledge, this study is the first to examine and compare transcriptome profiles during lentiviral infection in newborns and infants at different ages. Despite these strengths, this study had limitations. To ensure that all newborns and infants were infected with a single oral exposure to mimic peripartum exposure, the SHIV dose was possibly higher than the HIV dose a human infant might receive from exposure to maternal blood or vaginal secretions. Because the study was terminated early in infection, we cannot determine from our findings how virologic outcomes and disease severity may compare between age groups over a longer period of observation. Finally, we did not examine transcriptomes in gut and lymphoid tissues, the major sites of viral replication and host defense. Age-dependent dynamics of immunopathology at these anatomic sites should be elucidated in future studies.

Taken together, our findings suggest that rapid viral seeding combined with defective innate defenses, slow or defective adaptive responses, and elevated immune activation in newborns contribute to age-dependent differences in pathogenesis. We conclude that SHIV, like HIV, is more acutely damaging in newborns than in infants infected at older ages, reinforcing the validity of this model for understanding mechanisms of pathogenesis in vertically acquired HIV infection. Ultimately, therapeutic intervention as early as possible after birth is more likely to have a durable effect on disease progression by limiting damage to the immune system.

## Methods

### Sex as a biological variable.

Twelve newborn male and female outbred rhesus macaques of Indian origin (*Macaca mulatta*) were obtained from the breeding colony within days of birth, underwent veterinary evaluation, were adapted to bottle feeding in the Animal Biosafety Level 2 (ABSL-2) infant nursery, and were transferred to ABSL-2+ containment as soon as possible after 1 week of age. Sex was not considered as a biological variable. Both sexes of newborns were included in the study but were not balanced between groups because they were assigned over a period of 2 birthing seasons, and sex is random. The newborn group included 4 females and 2 males; the infant group included 2 females and 4 males. No differences could be attributed to sex, and we believe that the results are expected to be the same. Animals were excluded from the study if they possessed *Mamu*-B*08 and -B*17 MHC-I alleles (46, 47). Animals were housed as age-matched pairs and were monitored for clinical signs of disease by regular evaluation of body weight, peripheral lymph node size, appetite, behavior, and stool quality. Animals were euthanized under IACUC guidelines using standard methods consistent with the recommendations of the American Veterinary Medical Association Guidelines for Euthanasia (63).

### HepB immunization.

All animals received the HepB vaccine Recombivax HB (Merck) by intramuscular injection on the first day of the study (age 7–14 days). Infants received an additional dose at week 6.

### SHIV exposure.

A challenge stock of SHIV_SF162P3_ was generated in activated, CD4^+^-enriched macaque splenocytes inoculated with SHIV_SF162P3_ obtained from the AIDS Reagent Program (no. 6526). Virus production and in vitro and in vivo quantification methods are described in detail in an earlier publication (42). Each animal received a total of 2 mL (4.1 × 10^4^ TCID_50_, rhesus PBMCs) of undiluted, just-thawed, cell-free SHIV_SF162P3_ virus by swallowing while awake two 1 mL doses 10–15 minutes apart. Newborns were exposed to SHIV at 1–2 weeks of age (day 0 study time point). Infants were exposed at 15–16 weeks of age (week 14 study time point).

### Blood and tissue harvest and processing.

Peripheral blood was collected into EDTA blood tubes and centrifuged in the collection tubes at 750*g* for 30 minutes at 4°C with no brakes; plasma supernatant was stored at −80°C. The remaining blood fraction was resuspended in sterile PBS to double the original volume, and PBMCs were isolated by centrifugation in SepMate tubes (STEMCELL Technologies) over Lymphocyte Separation Medium (Corning) and cryopreserved in liquid nitrogen. At each biopsy, 2 adjacent LNs were taken to obtain a sample for paraffin embedding and to make a cell suspension that was cryopreserved. At necropsy, blood and CSF were collected into a 2 mL vial and stored at −80°C. Then 100 μg samples of solid tissues were excised and frozen at −80°C in 2 mL tubes prefilled with 1.4 mm zirconia beads (Spex SamplePrep) for tissue homogenization with a bead beater, for nucleic acid extraction and viral DNA detection by quantitative PCR. Any remaining spleen or mesenteric lymphoid tissue was processed to make single-cell suspensions and cryopreserved.

### Viral nucleic acid quantitation in plasma, cells, and tissue homogenates.

Nucleic acid from plasma, CSF, or lymph node biopsy cell pellets was purified using a Maxwell 16 instrument (Promega) per the manufacturer’s protocol, using the LEV Viral Total Nucleic Acid Kit for plasma and CSF and the LEV Whole Blood DNA Kit for lymph node cells. Complete PCR and reverse transcriptase PCR protocols for viral RNA, viral DNA in cell pellets, and viral DNA in tissues were published previously (42).

### Evaluation of pathology.

At necropsy, rhesus macaque tissues and body fluids were collected fresh, then frozen or fixed in 10% formalin and subsequently processed for histologic evaluation. Necropsies and microscopic evaluation of tissues were performed by veterinary pathologists masked to animal group assignments and viral load data. Pathogens were identified and confirmed by H&E morphology and histochemistry, IHC, and PCR. Adenovirus, CMV, and *Enterocytozoon bieneusi* were visualized by IHC using the VECTASTAIN ABC Kit, Peroxidase Standard (Vector Laboratories). Adenovirus was detected with a mouse anti-adenovirus mAb (MAB8052, 1:500 dilution, EMO Millipore). CMV was detected using an antibody gifted by Peter A. Barry, UCD School of Medicine (1:750 dilution), as previously reported (64). *E*. *bieneusi* were visualized using a mouse anti-measles matrix protein mAb (MAB8910, 1:1,000 dilution, EMO Millipore). *Spironucleus* species (sp) were confirmed in tissue by nested PCR amplification of small subunit ribosomal DNA using methods developed by Bailey et al. (65). *Cryptosporidium* sp, SHIV giant cell disease, flagellated protozoa, *Pneumocystis* sp, *Malassezia* sp, and attaching and effacing *E*. *coli* were all diagnosed by H&E stain. Additional diagnostic methods included Gomori methenamine-silver stain for *Pneumocystis* sp and periodic Schiff reaction for *Malassezia* sp. Intracellular argyrophilic bacteria were visualized by Warthin-Starry stain. Disease scores of 0, 1, or 2 were assigned to each animal according to the criteria outlined in Table 1.

### ELISA.

Antibody responses were determined by measuring binding of plasma IgG to recombinant HIV-1 SF162 gp140 trimer and described previously (42). The gp140 trimer was produced as described (66) by transient transfection in Expi293F cells and purified over a *Galanthus nivalis* lectin-coupled agarose column (Vector Laboratories), followed by size-exclusion chromatography on a Superdex 200 column (GE Healthcare Life Sciences, now Cytiva). Peak trimer fractions were pooled, concentrated, and frozen at −20°C.

### Env-pseudovirus construction and neutralization assay.

SHIV gp160/pEMC* vector DNA was cotransfected with pSG3ΔEnv HIV-1 backbone DNA into HEK293T cells (European Collection of Authenticated Cell Cultures, MilliporeSigma catalog number 12022001) using the jetPEI (Polyplus) transfection reagent. Pseudovirus stocks were titrated in TZM-bl cells (NIH AIDS Reagent Program, no. 8129) to determine the virus dilution required for 200,000 relative light units. Heat-inactivated plasma samples were assayed in duplicate for neutralization against a single round of entry of SHIV_SF162P3_ Env-pseudoviruses using TZM-bl reporter cells.

### ELISPOT.

T cell responses in blood and tissues were tested in an IFN-γ ELISPOT assay as previously described (42).

### Flow cytometry.

To evaluate CD8^+^ T cells and monocytes in blood and tissues, 2 staining panels were developed, and samples were run on a Becton-Dickinson LSR II flow cytometer. Data were analyzed in FlowJo according to the gating strategies defined in Supplemental Figures 3 and 4 for panels 1 and 2, respectively.

### RNA isolation and deep sequencing (RNA-Seq).

From 500 μL peripheral whole blood collected in EDTA tubes, total cellular RNA was isolated using the QIAamp RNA Blood Mini Kit (QIAGEN, catalog number 52304). RNA was eluted in 30 μL nuclease-free water, split into 2 aliquots of 23 μL and ~5 μL, and immediately frozen at –80°C. Frozen RNA aliquots were shipped on dry ice to MedGenome for sample quality control, barcoded cDNA library construction, and bulk deep sequencing (RNA-Seq) using the Illumina platform. Barcoded cDNA libraries were built using the Illumina TruSeq Stranded Total RNA kit with globin removal. Sequencing was performed with an Illumina NovaSeq instrument using a 100 bp paired-end read protocol for a read depth of ~50 million total reads (~25 million paired end reads) per sample. A total of 56 samples were processed to generate 56 read sets, of which 54 were used in downstream transcriptome analysis. The remaining 2 samples were included as backups for animal 38371 CNB42DPB that had low RNA quality. The sample in question successfully yielded a barcoded library and good quality sequence data, and backup read sets were not analyzed further.

### RNA-Seq computational pipeline and statistical analysis.

RNA-Seq read sets were analyzed for read quality, then aligned to the rhesus Mmul10 reference genome, and genes were enumerated by the STAR method. Downstream processing of the count matrix data was performed in R (3.6.3) (67). Data were normalized with the centered log-ratio method (68), and DE between contrasts of interest was analyzed using DESeq2 (69). Contrasts of interest are summarized in Figure 8A and Supplemental Figure 5. Genes were considered significantly differentially expressed if adjusted *P* < 0.1 using the FDR method to correct for multiple comparisons. Only significantly differentially expressed genes were displayed in the heatmaps and included in the transcriptomic signatures for each contrast. To increase power to detect differences, direct comparisons between groups were performed without setting up multifactor models, because the study was designed to control for the potentially confounding factors of sex, age, and environment. Pairwise-clustered heatmaps were generated using the pheatmap package using complete clustering on Euclidean distances (70). Venn diagrams were constructed using the VennDiagram package (71) to visualize differentially expressed gene sets that overlap between different contrasts. For Venn diagrams, genes were considered significantly differentially expressed if adjusted *P* < 0.1. To obtain insight into biological functions and pathways of differentially expressed gene signatures, we used GeneSetVis, a custom-built bioinformatics tool, to mine the MSigDB, StringDB (GO and KEGG), and Reactome databases for functional annotation of significant genes, to identify pathways and cellular processes that are differentially expressed in contrasts of interest. Significantly enriched terms (adjusted *P* < 0.1) that were represented by at least 3 query genes were reported in Figure 8 and Supplemental Figure 6.

### Statistics.

Newborn rhesus macaques were assigned randomly to the newborn or infant study groups as they accrued, regardless of sex. Historical studies using this animal model showed that group sizes of 6 were sufficient to provide 80% power to detect a 1-log difference in plasma viral loads. For viral quantitation in plasma and tissues, measurement of T cell responses by ELISPOT, and pathology evaluation, experimenters were masked to the animal group assignments. No masking was possible for longitudinal flow cytometry experiments, transcriptome analysis, and ELISAs. Nonparametric tests were used to compare groups for viral loads in plasma and tissues, and immunological metrics as measured by flow cytometry and ELISA. The specific statistical approach used for each hypothesis test is noted in the figure legends. *P* values less than 0.05 were considered significant.

### Study approval.

Macaque studies were performed at the Oregon National Primate Research Center (ONPRC) in Beaverton, Oregon, USA, in compliance with all ethical regulations for animal testing and research. The ONPRC is accredited by Association for Assessment and Accreditation of Laboratory Animal Care International and adheres to the Guide for the Care and Use of Laboratory Animals and the US Public Health Service Policy on the Humane Care and Use of Laboratory Animals. The study protocol was approved by the Oregon Health & Science University (OHSU) West Campus Institutional Animal Care and Use Committee.

### Data availability.

Supporting data are provided in an accompanying Supporting Data Values Excel file. Raw RNA-Seq data have been uploaded to the NIH Sequence Read Archive database, BioProject PRJNA640434, and analyses can be found at https://doi.org/10.5281/zenodo.11459851

## Author contributions

MBS, BB, JBS, AJH, and NLH designed the experiments. MBS optimized SHIV stock production, designed and performed the flow cytometry experiments, isolated RNA, and analyzed and interpreted data. TO managed tissue collection and databases, isolated RNA, analyzed virology data, and performed ELISA and neutralization assays. SP coordinated animal assignments, treatments, and procedures. MBS and SP generated and titrated the SHIV_SF162P3_ stock used for newborns and infants. EM performed RNA-Seq bioinformatics analysis and made heatmaps. KO developed and used the GeneSetVis program to facilitate GO and pathway enrichment analysis of transcriptomic datasets. JR performed ELISPOT assays. HS provided veterinary care and advice for animals. JS performed biopsies and advised on surgical procedures. LMC, AJ, and ADL performed necropsies and described pathology. All authors discussed the results. MBS and NLH wrote the manuscript. NLH supervised the research.

## Supplementary Material

Supplemental data

Supporting data values

## Figures and Tables

**Table 1:**
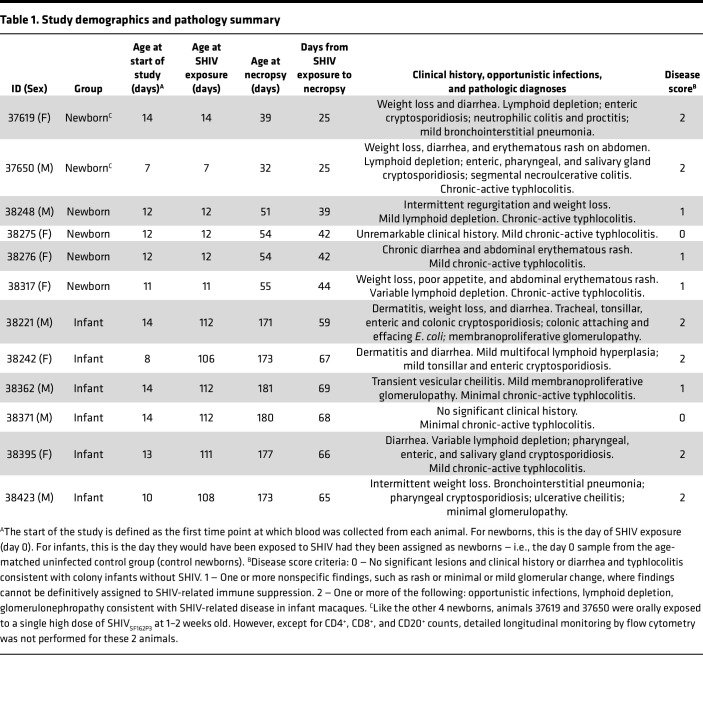
Study demographics and pathology summary

## Version 1 08/27/2024

In-Press Preview

## Version 2 10/08/2024

Electronic publication
