# Supplementary material for: Immune perturbation following SHIV infection is greater in newborn macaques than in infants
Source: JCI Insight. 2024 Aug 27;9(19):e144448. doi: 10.1172/jci.insight.144448 (PMC11466190; doi:10.1172/jci.insight.144448)
Supplement: Supplemental data [file jciinsight-9-144448-s157.pdf]

Shapiro, et al. **Immune Perturbation Following SHIV Infection is Greater in Newborn Macaques Than in Infants**

Supplemental Table 1. Tissue viral loads per million cells, log transformed

Tissues[1] and Group	Animal numbers					
**Newborns**	**37619**	**37650**	**38248**	**38275**	**38276**	**38317**
Axillary LN	4.449	4.760	4.913	4.869	4.814	4.724
Mixed Mesenteric LN	4.939	5.105	4.953	4.876	4.871	5.085
Iliosacral LN	4.597	4.799	5.150	4.788	4.810	4.806
Inguinal LN	4.573	4.814	5.014	4.939	4.889	4.903
Spleen	4.133	4.266	4.644	4.413	4.477	4.426
Duodenum	4.160	4.383	3.301	3.212	3.347	3.264
Jejunum	4.087	3.421	4.357	2.809	2.905	3.141
Ileum	3.995	4.216	3.910	3.878	4.025	3.383
Cecum	3.660	3.852	4.052	3.576	4.095	3.790
Colon Descending	3.675	3.699	4.166	3.519	4.097	3.862
Rectum	3.567	3.709	3.872	4.751	3.772	4.269
**Infants**	**38221**	**38242**	**38362**	**38371**	**38395**	**38423**
Axillary LN	4.845	3.646	4.488	4.349	4.426	4.502
Mixed Mesenteric LN	4.800	3.963	4.827	4.560	4.701	4.663
Iliosacral LN	4.759	3.873	4.434	4.365	4.709	4.072
Inguinal LN	4.809	3.875	4.526	4.398	4.561	4.426
Spleen	4.702	3.310	4.049	3.880	4.350	3.660
Duodenum	3.741	3.291	3.843	3.746	3.020	4.023
Jejunum	3.652	2.857	2.949	3.536	2.621	3.353
Ileum	3.923	3.696	3.883	3.724	3.714	3.710
Cecum	3.428	3.812	3.743	3.601	3.051	3.217
Colon Descending	3.569	3.348	3.893	n.d. [2]	3.805	3.763
Rectum	3.442	2.229	3.913	3.459	3.256	3.513
**Adults**	**27524**	**35152**	**35533**	**36571**	**35529**	**35719**
Axillary LN	2.885	2.492	2.906	2.960	3.944	3.591
Mixed Mesenteric LN	3.522	3.371	3.508	2.733	n.d. [2]	3.813
Iliosacral LN	2.839	3.296	2.727	2.359	3.996	3.633
Inguinal LN	3.294	3.408	3.067	2.691	4.114	3.580
Spleen	3.111	3.310	2.645	1.970	3.813	3.380
Duodenum	2.275	2.083	1.645	1.635	3.041	2.740
Jejunum	2.069	1.698	1.968	0.880	2.869	2.362
Ileum	2.268	2.495	2.845	1.893	2.716	2.204
Cecum	2.231	2.434	2.385	1.880	3.556	2.813
Colon Descending	2.345	2.528	2.621	1.818	3.301	3.041
Rectum	2.349	2.538	2.217	1.973	3.431	3.176

[1]Tissues analyzed by qPCR for DNA copy number included axillary LN, mixed mesenteric LN, iliosacral LN, Inguinal LN for lymphoid tissues; duodenum, jejunum, ileum, cecum, colon, and rectum for gut tissues; and spleen.
[2]n.d. no sample

Supplemental Table 2
Mixed Effects Model on Log Transformed Viral Copy Numbers in Eleven Tissues

Tissues analyzed at necropsy[1]	Contrast n=6 per group[2]	Fold change	Lower bound	Upper bound	P value	Bonferroni adjusted P value
All 11 tissues	Infant vs adult	1.9896	0.825	4.691	0.126783	1.00000
	Newborn vs adult	4.237	1.778	10.009	***0.001119***	***0.01343***
	Infant vs newborn	0.464	0.195	1.107	0.083406	1.00000
Four lymphoid tissues	Infant vs adult	2.171	0.866	5.444	0.098384	1.00000
	Newborn vs adult	5.591	2.230	14.022	***0.000243***	***0.00292***
	Infant vs newborn	0.388	0.155	0.972	***0.043243***	0.51891
Six gut tissues	Infant vs adult	1.955	0.801	4.774	0.141097	1.00000
	Newborn vs adult	3.641	1.492	8.883	***0.004516***	0.05420
	Infant vs newborn	0.537	0.220	1.311	0.172271	1.00000
Spleen	Infant vs adult	1.379	0.450	4.227	0.573820	1.00000
	Newborn vs adult	3.473	1.133	10.646	***0.029358***	0.35229
	Infant vs newborn	0.397	0.130	1.217	0.106047	1.00000

[1]Tissues analyzed by qPCR for DNA copy number included axillary LN, mixed mesenteric LN, iliosacral LN, Inguinal LN for lymphoid tissues; duodenum, jejunum, ileum, cecum, colon, and rectum for gut tissues; and spleen. See **Supplemental Table 1** for the data.
[2]Mean difference in the form of fold change using log transformed DNA copy numbers was performed prior to regression analysis, 95% confidence intervals (lower bounds and upper bounds), P value and Bonferroni adjusted P values are shown.

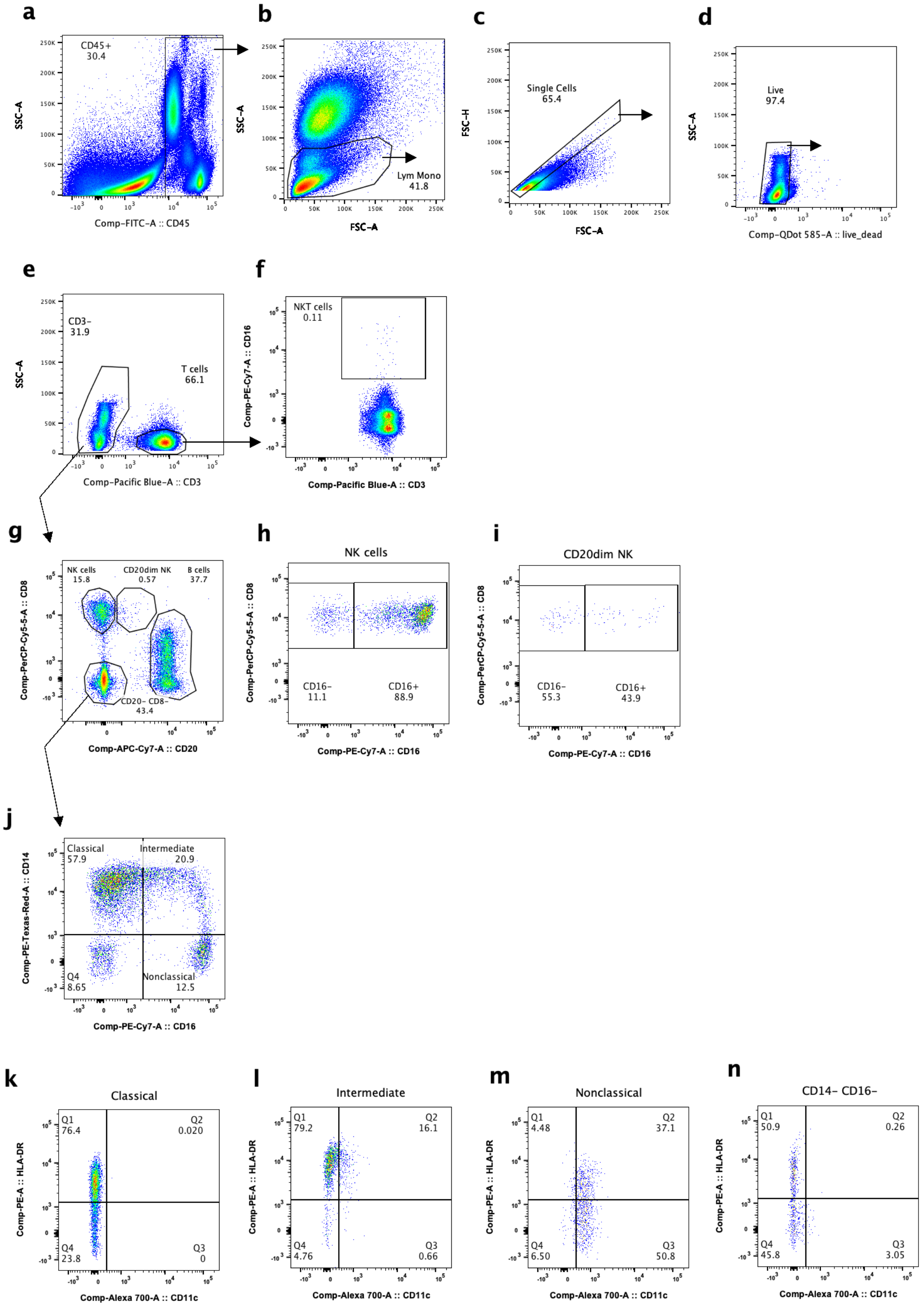

Supplemental Figure 1. Flow cytometry gating strategy for Panel 1 (B cells, NK cells, NKT cells, monocytes). Populations of interest were analyzed after gating on CD45+ **(a)** lymphocytes and monocytes by forward and side scatter profile **(b)**, then excluding doublets **(c)** and dead cells **(d)**. T cells were defined as CD3+ **(e)**, and NKT cells were defined as CD3+ CD16+ **(f)**. Within the CD3- gate, B cells as well as two populations of NK cells (CD20- and CD20dim) were defined based on CD20 and CD8 expression **(g)**. In some animals, including the representative sample shown, a sizeable fraction of CD20+ cells expressed CD8. Follow-up experiments showed that these CD20+ CD8+ cells were true singlets; stained positive with

antibodies to different CD8 epitopes; were negative for NKG2A, CD56, and CD8b, and positive for CD19 and CD79a; and were present at remarkably constant frequencies specific to individual animals over time. Based on these findings, we considered them B cells and included them in the absolute B cell count. Expression of CD16 was measured on **h)** CD20- NK cells and **i)** CD20dim NK cells. Within the CD8- CD20- population, which was comprised primarily of monocytes, three monocyte subsets were defined based on their expression of CD14 and/or CD16; cells in the double-negative gate likely included dendritic cells and basophils, and were not analyzed further **(j)**. Expression of CD11c and HLA-DR was evaluated in each monocyte subset **(k-m)** and in the double-negative population **(n)**. Note that HLA-DR was not used in the gating strategy for monocytes because replacement and recalibration of the green laser during the study resulted in inconsistent signal strength for HLA-DR PE during the longitudinal sampling period. Each blood sample was collected, stained, and analyzed individually. Antibody stain mixes and flow cytometer voltage settings were kept the same for the duration of the study. Gates were drawn based on obvious divisions between positive and negative populations, where possible; if the separation of populations was indistinct, positive cutoffs were determined based on a cell subset known to be negative for the marker in question.

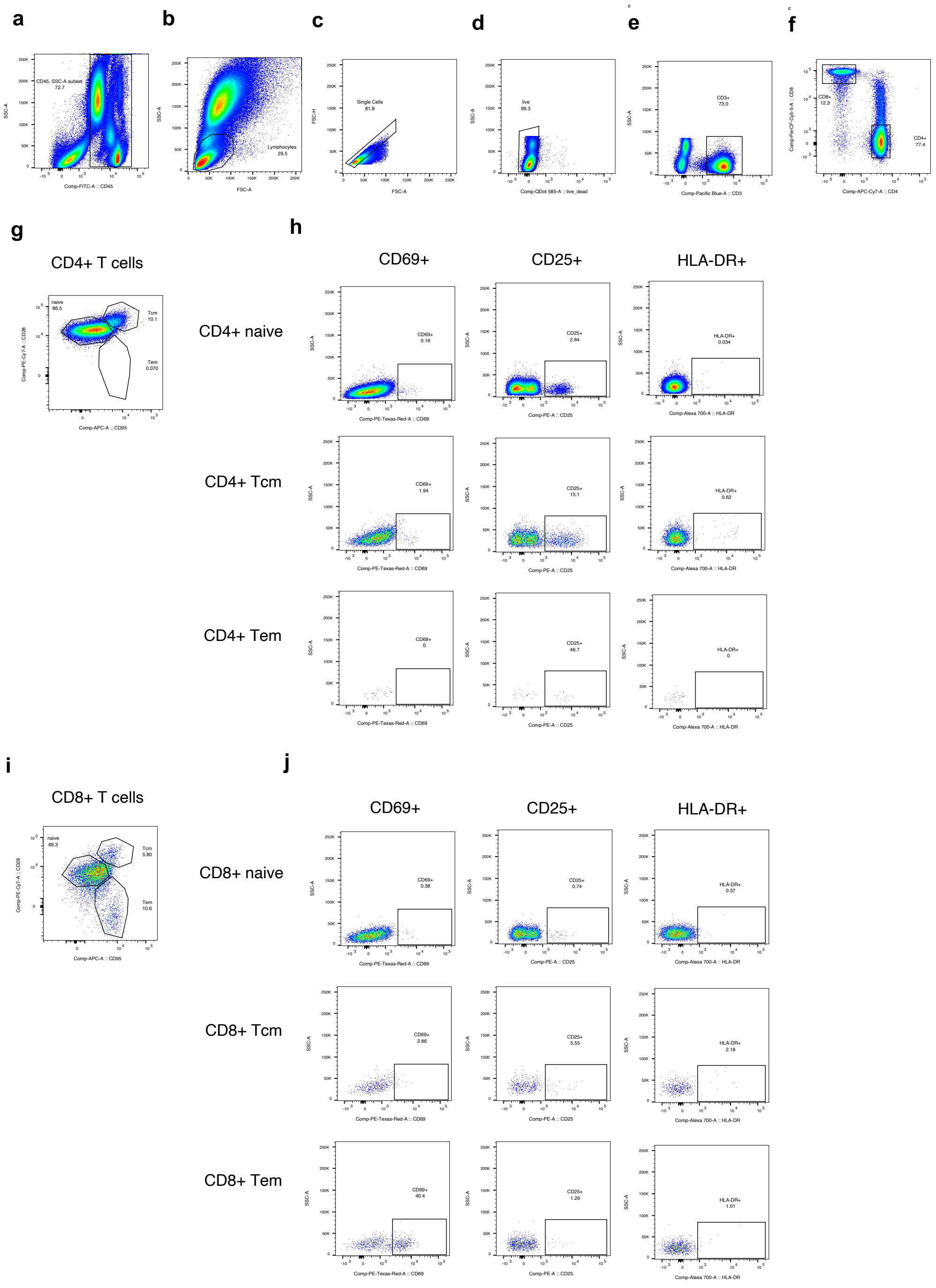

Supplemental Figure 2. Flow cytometry gating strategy for Panel 2 (T cells). Populations of interest were analyzed after gating on CD45+ cells **(a),** then lymphocytes by forward and side scatter profile **(b)**, then excluding doublets **(c)** and dead cells **(d)**. T cells were defined as CD3+ **(e)**, and within the CD3+ gate, CD4+ and CD8+ T cells were discriminated **(f)**. CD4+ T cells were further subdivided into naïve, central memory (Tcm), and effector memory (Tem) subsets on the basis of CD28 and CD95 expression **(g)**. Each of these subsets was evaluated for expression of the activation markers CD69, CD25, and HLA-DR **(h)**. The same strategy was applied to the analysis of CD8+ T cell subsets and their activation marker profiles **(h-i)**. Each blood sample was collected, stained, and analyzed individually. Gates were drawn based on clear divisions between positive and negative populations, where possible. If the separation of

populations was indistinct, positive and negative cutoffs were determined based on a cell subset known to be negative for the marker in question.

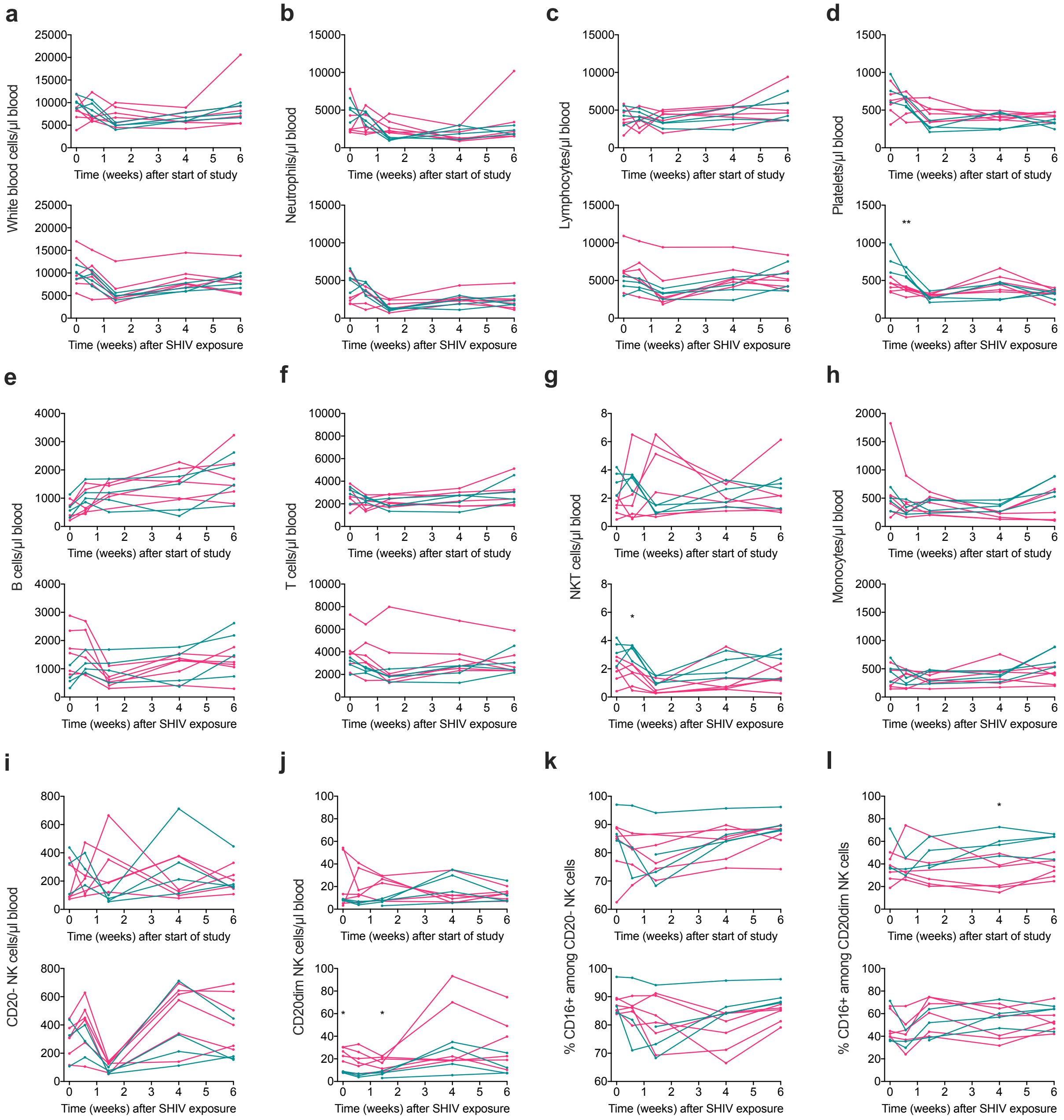

Supplemental Figure 3. Major leukocyte populations in newborns and infants before and during SHIV infection. Leukocyte populations in blood were monitored longitudinally by flow cytometry and complete blood count (CBC). Newborns are shown in teal and Infants (or Control Newborns) in pink. For each set of graphs, the top graph shows measurements after the start of the study, so any differences between the groups are due to SHIV infection status (teal: SHIV+ Newborns, pink: SHIV- Control Newborns). The bottom graph shows measurements after SHIV infection, so any differences between the groups are due to age at the time of SHIV exposure (teal: Newborns at 1-2 weeks, pink: Infants at 15-16 weeks). **a)** Absolute white blood cell counts. **b)** Absolute neutrophil counts. **c)** Absolute lymphocyte counts. **d)** Absolute platelet counts. **e)** Absolute B cell counts. **f)** Absolute T cell counts. **g)** Absolute NKT cell counts. **h)** Absolute monocyte counts. **i)** Absolute counts of CD20- NK cells. **j)** Absolute counts of CD20dim NK cells. **k)** Percentages of CD20- NK cells that express CD16. **l)** Percentages of CD20dim NK cells that express CD16. Pairwise statistical comparisons at each time point were performed using Sidak's multiple comparisons test. *, $p < 0.05$. **, $p < 0.01$.

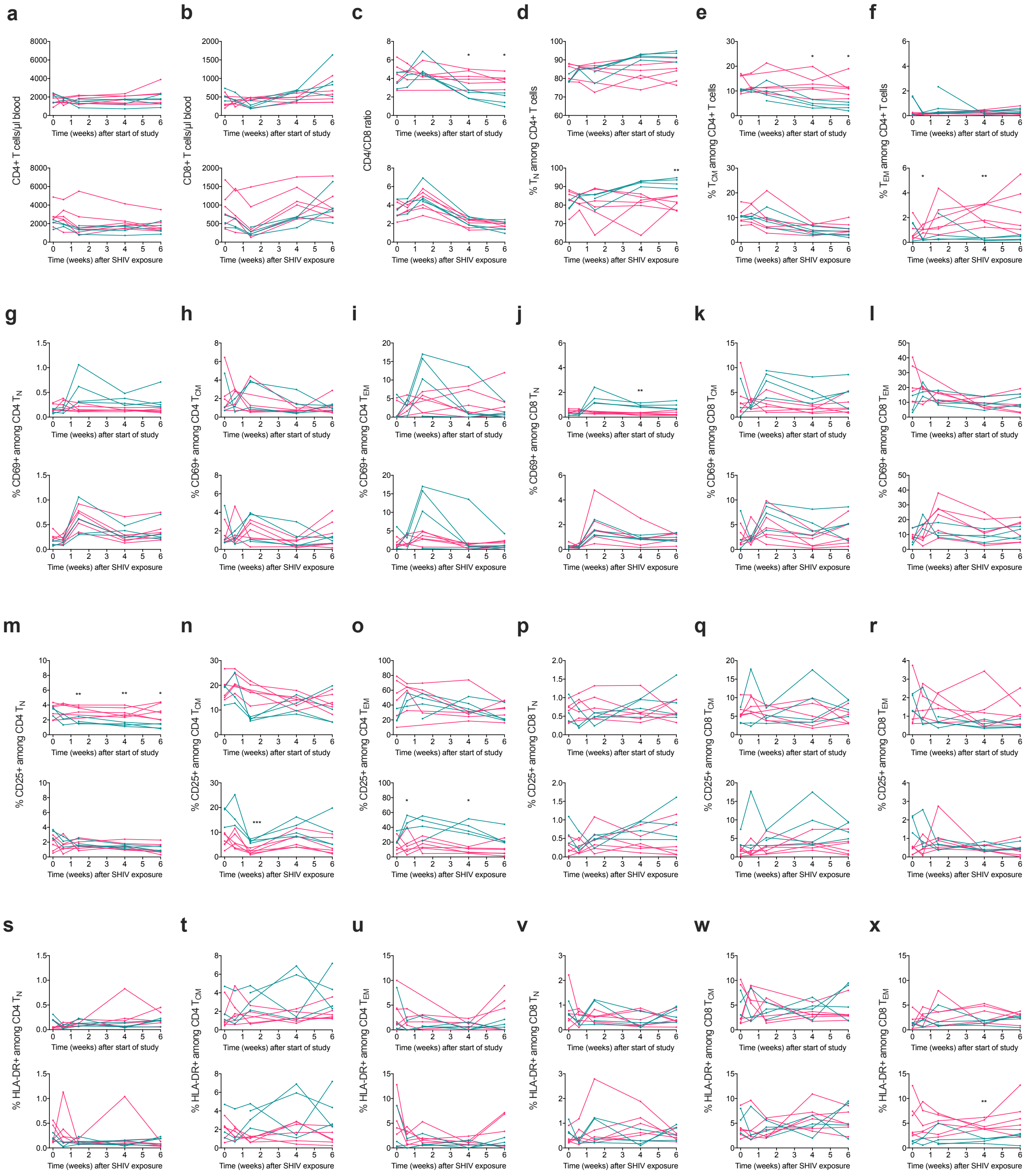

Supplemental Figure 4. T cell counts, CD4+ subsets, and activation phenotypes in newborns and infants before and during SHIV infection. Peripheral blood T cell populations and activation markers were monitored longitudinally by flow cytometry. Newborns are shown in teal and Infants (or Control Newborns) in pink. For each set of graphs, the top graph shows measurements after the start of the study, so any differences between the groups are due to SHIV infection status (teal: SHIV+ Newborns, pink: SHIV- Control Newborns). The bottom graph shows measurements after SHIV infection, so any differences between the groups are due to age at the time of SHIV exposure (teal: Newborns at 1-2 weeks, pink: Infants at 15-16 weeks). **a)** Absolute counts of CD4+ T cells. **b)** Absolute counts of CD8+ T cells. **c)** Ratio of CD4+ to CD8+ T cells. **d)** Percentages of naïve CD4+ T cells (CD28+ CD95-). **e)** Percentages of central memory CD4+ T cells (CD28+ CD95+). **f)** Percentages of effector memory CD4+ T cells (CD28-

CD95+). **g-i)** Percentages of naïve, central memory, and effector memory CD4+ T cells expressing CD69. **j-l)** Percentages of naïve, central memory, and effector memory CD8+ T cells expressing CD69. **m-o)** Percentages of naïve, central memory, and effector memory CD4+ T cells expressing CD25. **p-r)** Percentages of naïve, central memory, and effector memory CD8+ T cells expressing CD25. **s-u)** Percentages of naïve, central memory, and effector memory CD4+ T cells expressing HLA-DR. **v-x)** Percentages of naïve, central memory, and effector memory CD8+ T cells expressing HLA-DR. Pairwise statistical comparisons at each time point were performed using Sidak's multiple comparisons test. *, $p < 0.05$. **, $p < 0.01$. ***, $p < 0.001$.

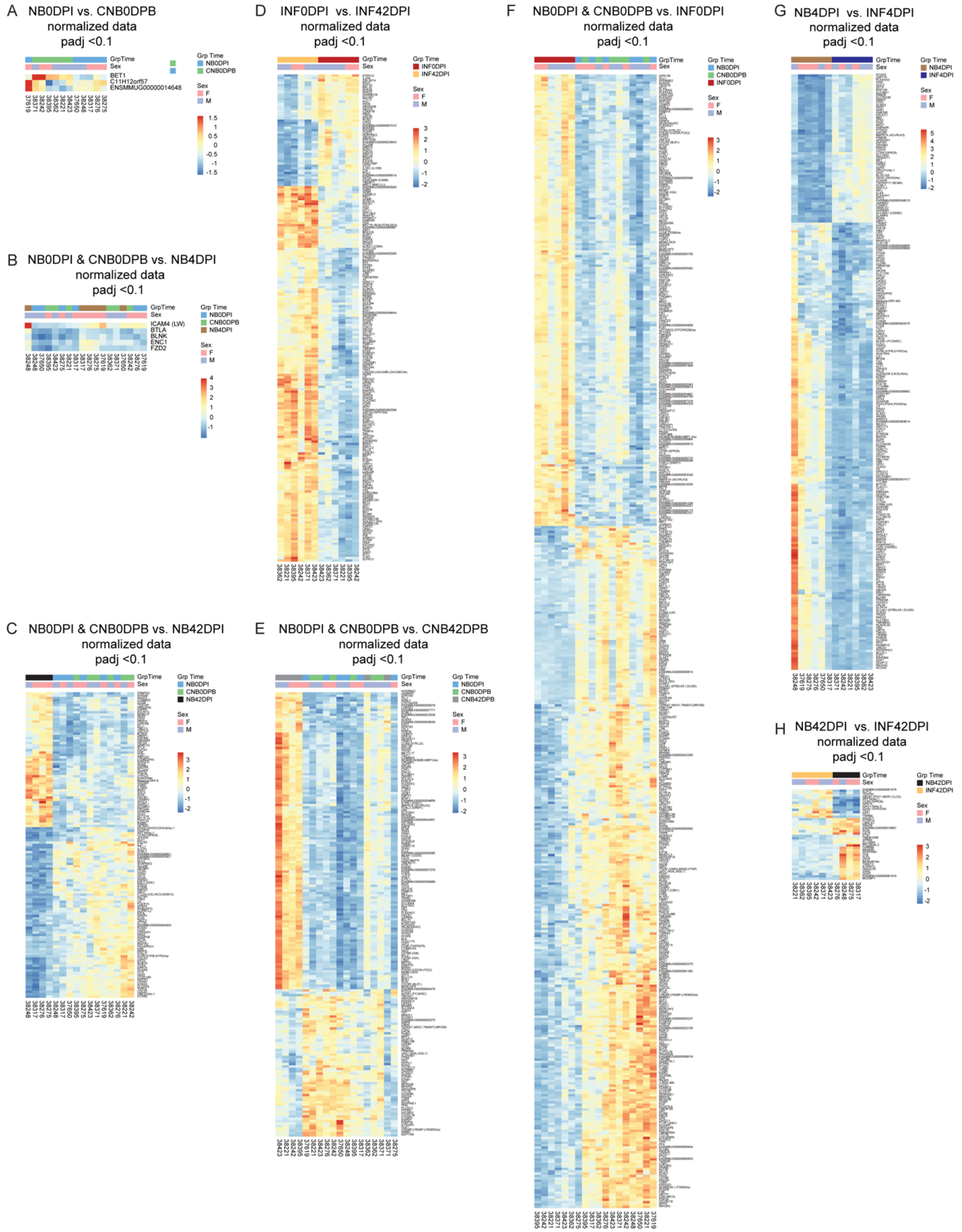

Supplemental Figure 5. Transcriptional signatures in background and between-group contrasts. Differentially expressed (DE) genes shown as heatmaps, with data normalized for P values adjusted to <0.1 DPB indicates days post beginning and DPI indicates days post infection. **A)** DE genes in Newborns vs. Control Newborns on study day 0. The only significantly DE genes were BET1 (higher in Control Newborns), which encodes a Golgi vesicular trafficking

protein; C11H12orf57 (higher in Newborns), which encodes the ubiquitously expressed C10 protein that is involved in brain development; and ENSMMUG00000014648 (higher in Newborns), coding for 40S ribosomal protein S18. **B)** DE genes in combined Newborn and Control Newborns (0 $DPB_{pooled)}$ vs. Newborns at 4 DPI. **C)** DE genes in combined Newborns and Controls (0 DPB_{pooled}) vs. Newborns at 42 DPI **D)** DE genes in Infants at 0 DPI vs. Infants at 42 DPI. **E)** 0 DPB_{pooled} vs. Control Newborns at 42 DPB, prior to infection. **F)** DE genes DPB_{pooled} vs. Infants at 0 DPI, which is study day 98, or 98 DPB **G)** DE genes at 4 DPI in Newborns vs. Infants. **H)** DE genes at 42 DPI in Newborns vs. Infants.

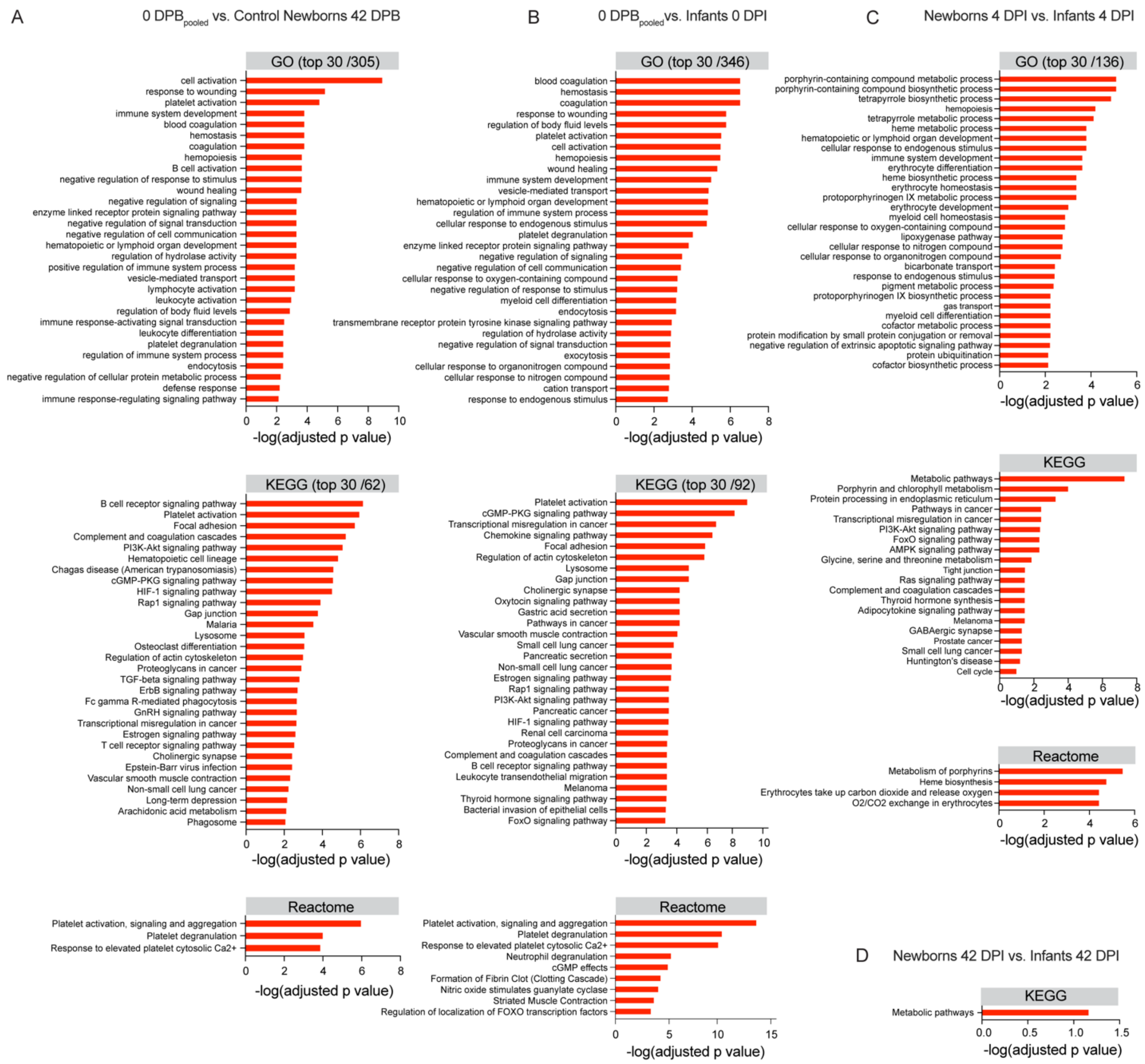

Supplemental Figure 6. GO, KEGG, and Reactome analyses of Differentially Expressed Genes. a) GO, KEGG, and Reactome analyses of DE genes in Control Newborns at 42 DPB vs. 0 DPB_{pooled}. Where many terms were significant (adjusted p < 0.1), top significant terms are shown. **b)** GO, KEGG, and Reactome analyses of DE genes in Infants at 0 DPI—which is also study day 98 (98 DPB)—vs. 0 DPB_{pooled}. Where many terms were significant (adjusted p < 0.1), top significant terms are shown. **c)** GO, KEGG, and Reactome analyses of DE genes at 4 DPI in Newborns vs. Infants. Where many terms were significant (adjusted p < 0.1), top significant terms are shown. **d)** KEGG analysis of DE genes of DE genes at 42 DPI in Newborns vs. Infants (adjusted p < 0.1). No significant terms were found in GO or Reactome analyses.
